# Environmental complexity shapes maintenance of bacterial diversity through context-dependent interactions among niche axes

**DOI:** 10.1093/ismejo/wrag144

**Published:** 2026-06-11

**Authors:** Jessica L Gronniger, Denisse Larin-Henriquez, Jessica R Bernardin, Lauren G Shoemaker, Leonora S Bittleston

**Affiliations:** Department of Biological Sciences, Boise State University, Boise, ID, 83725-1512, United States; Department of Biological Sciences, Boise State University, Boise, ID, 83725-1512, United States; Department of Biological Sciences, Boise State University, Boise, ID, 83725-1512, United States; Department of Botany, University of Wyoming, Laramie, WY, 82071, United States; Department of Biological Sciences, Boise State University, Boise, ID, 83725-1512, United States

**Keywords:** microbial coexistence, synthetic community, niche availability, niche complexity, diversity, synergy

## Abstract

Microbial communities are often more species-rich than predicted from classical ecological models. The high levels of coexistence observed in nature are typically attributed to forces that modulate niche availability and stabilize communities. Specific drivers of niche partitioning are often tested in isolation, and the interactive effects of niche variation across resources, space, and time have not been tested together experimentally to determine how they affect community responses. Here, we used 26 bacterial strains previously isolated from carnivorous pitcher plant (*Sarracenia purpurea*) aquatic pools to construct and expose species-rich synthetic communities to four factors that alter environmental complexity in a fully factorial design, creating combinations of resource complexity, spatial niche structure, and temporal fluctuations. Across treatments, increased niche complexity generally, but not always, promoted the long-term retention of more species, with a saturating effect at the highest levels of complexity. Resource complexity emerged as a primary driver of diversity, with its effects also depending on other niche axes. Interactions among factors frequently deviated from additive expectations, with both synergistic and antagonistic effects observed depending on the combination of conditions. Together, these results show that environmental complexity shapes bacterial diversity through context-dependent, nonlinear interactions among niche dimensions, highlighting that the relationship between niche dimensionality and diversity is contingent on how environmental factors combine.

## Introduction

Ecological communities are often more species-rich than expected from traditional theory [1–3], with high levels of coexistence observed in plant [4, 5], animal [6], and microbial assemblages [7] despite competition and limited niche space. This discrepancy between classical model predictions and observed diversity prompted the development of modern coexistence theory, which suggests that coexistence occurs when mechanisms allowing species to differentiate in niche space are sufficient to stabilize against inherent differences in fitness between species that would otherwise result in competitive exclusion [7–9]. These stabilizing niches—which can be separated into four primary axes: resources, time, space, and natural enemies [8]—have been shown to promote coexistence in natural communities. For example, several species of Australian songbirds with significant niche overlap coexist due to highly specialized foraging behaviors [10]; overlapping tundra plant species in Alaska coexist by partitioning differentially available forms of nitrogen [11]; and ecologically similar species of Brocket deer coexist through temporal and habitat partitioning [12]. While the effects of these niche axes have been examined empirically, studies thus far have occurred primarily in plant systems [13–15]. Fewer experiments have attempted to directly measure their effects for microbial diversity, despite microbial communities often sustaining higher diversity with dozens to thousands of microbial species co-occurring, even on very small scales. Given that microbes make up the majority of Earth’s biodiversity [16], harbor the majority of life’s metabolic capabilities [17–19], and are essential to most multicellular organisms and ecosystems [20, 21], it is critical to understand if theory originally developed for macro-systems also predicts the maintenance of microbial diversity.

It is particularly challenging to identify the mechanisms driving the assembly and coexistence of species-rich natural microbial communities due to their high complexity, difficulties in directly quantifying species interactions, and the many biotic (e.g. competition and cross-feeding) and abiotic (e.g. environmental conditions and fluctuations) factors affecting community composition and function. However, synthetic microbial communities, where individual isolates are added together in controlled laboratory experiments, have the potential to further this research [22]. Several studies using simple microbial communities have hinted at how individual niche axes can drive coexistence. For example, metabolic leaking and cross-feeding [23], microscale spatial structure [24], spatiotemporal niche partitioning [25], dormancy [26], and cooperative growth [27] have all led to higher-than-expected levels of coexistence. Multiple studies have also identified a significant relationship between diversity and the availability and complexity of resources [28, 29], although the nature of this relationship remains unclear. The relationship between microbial diversity and resource availability is nuanced, with resource abundance vs. complexity often having different outcomes. Higher microbial diversity can arise in nutrient-limited conditions [30, 31], whereas increased nutrient complexity largely fosters greater diversity by reducing competition, promoting resource specialization [32], and increasing the prevalence of cross-feeding among community members [33, 34]. The resource–diversity relationship is likely dependent not only on the complexity of resources available [28] but also on the composition of the microbial community in question and members’ ability to utilize and partition those resources through mechanisms that expand niche space such as metabolic niche construction, cross-feeding [35, 36], resource partitioning, and metabolic switching [37]. Together, the evidence suggests that increasing spatial, temporal, and resource diversity likely leads to higher overall community richness and associated increases in ecosystem function. However, while current research has elucidated the role of some individual axes in promoting coexistence, different types of mechanisms have rarely been tested together experimentally to evaluate how they act in concert.

Recent studies investigating the interactive effects of multiple niche axes mostly use plant and animal systems and are often limited to two interacting axes while relying heavily on computational models rather than experimental data. For example, models applied to simulated perennial [38] and annual [39] plant dynamics found that interacting mechanisms of coexistence, such as relative nonlinearity, storage effects, and frequency-dependent seed predation, contributed to coexistence in a highly contextual manner [40]. These studies measured both subadditive and synergistic effects on coexistence, particularly in fluctuating environments [38, 39]. Similar results were found using nectar yeasts, where experiments and simulations suggested that both relative nonlinearity and storage effects independently contributed to coexistence; however, interactions between these mechanisms were both antagonistic (subadditive) or complementary (synergistic), with resulting effects often depending on additional environmental context [41]. While simulations [42–44] and limited experimental data [7, 15, 45] have hinted at the highly interactive and contextual nature of co-occurring mechanisms of coexistence, to our knowledge, no study has simultaneously tested the effect of more than two axes of niche space on coexistence. Here, we attempt to address this knowledge gap by utilizing a species-rich synthetic community (composed of 26 distinct isolates) to better represent dynamics in highly diverse natural communities and exposing this diverse community to gradients of environmental complexity designed to simultaneously test interactions between four distinct axes of niche space.

**Figure 1:**
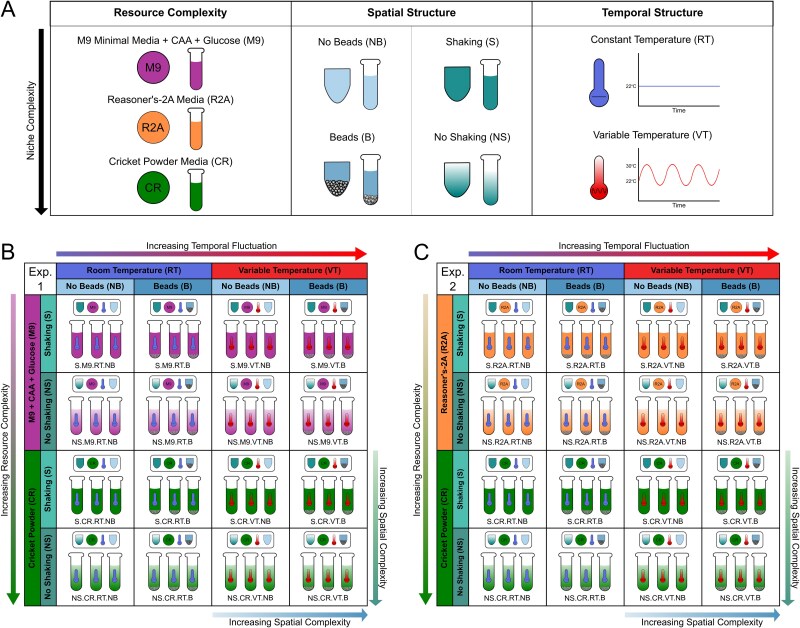
Experimental design. In two parallel, independent experiments, synthetic microbial communities were exposed to four distinct environmental conditions and all their factorial combinations. (A) Treatments were selected to represent a range of resource (media type), spatial (beads versus no beads, shaking versus no shaking), and temporal (constant versus fluctuating temperature) niche complexity. Although comparable in their design, the two experiments primarily differed in the type of low-complexity media utilized, with the first experiment (B) using M9 + CAA+ glucose whereas the second experiment (C) used R2A, both of which were compared to the relatively more complex cricket powder media (CR). Due to M9 not supporting growth of one isolate, Experiment 1 used 25 bacterial isolates, whereas Experiment 2 used 26 isolates. In both experiments, three replicates of the respective constructed communities were assigned to each of the 16 factorial treatment combinations. Communities were transferred into fresh media at a 1:5 ratio every 3 days for a total of 12 transfers (39 days total). In panels (B) and (C), each experimental condition is labeled according to their respective environmental conditions; for example, “NS.CR.VT.B” represents the no shaking (NS), cricket media (CR), variable temperature (VT), and beads (B) treatment, whereas “S.M9.RT.NB” represents the shaking (S), M9 + CAA+ glucose media (M9), constant room temperature (RT), and no beads (NB) treatment.

To measure how niche variation across resources, space, time, and their combined effects impact the diversity of complex multispecies bacterial communities under controlled conditions, we constructed and exposed a synthetic bacterial community to four treatments that manipulated different aspects of environmental variability and all their factorial combinations. We used bacterial strains previously isolated from *Sarracenia purpurea*, a carnivorous pitcher plant that is a well-established model for community ecology [46–48], to construct a 26-species synthetic community. We then ran two independent experiments where the synthetic community was exposed to four treatments in a fully factorial design intended to vary resource complexity, spatial structure, and temporal fluctuations. Treatments included (i) the use of two bacterial media types (with lower vs. higher resource complexity), (ii) exposure to gentle shaking or static (no shaking) conditions, (iii) the addition or absence of 0.5 mm glass beads, and (iv) incubation at either constant room temperature (~22°C) or variable temperatures fluctuating between 22°C and 30°C on a 6-h cycle. Variation in resource complexity was created with the different media types, physical niche space availability with shaking to homogenize conditions versus no shaking (allowing for more stratification) and adding beads (increasing physical structure and thus creating spatial heterogeneity) versus no beads, and temporal fluctuations with variable versus constant temperatures. For each of the two independent experiments, we assigned three replicates of our synthetic communities to each of the 16 factorial combinations of treatments and passaged them every 3 days for a total of 39 days (Fig. 1). By exposing a complex synthetic microbial community to a combination of different axes of niche complexity and measuring the resulting diversity, we tested two main hypotheses. We hypothesized that (i) there would be a positive overall relationship between niche complexity and multispecies diversity, where the creation of additional niches in both space and time would promote coexistence, and (ii) different axes of niche space would have synergistic interactions that support increased diversity by creating additional niches through interacting environmental conditions. Beyond these two hypotheses, we also sought to explore potential mechanisms underlying any observed relationship between diversity and niche complexity by investigating strain-level responses and metabolic potential.

## Materials and methods

We used 26 bacterial strains previously isolated from carnivorous pitcher plant (*S. purpurea*) aquatic pools to construct and expose species-rich synthetic communities to four factors that altered environmental complexity in a fully factorial design, creating combinations of resource complexity, spatial niche structure, and temporal fluctuations intended to investigate the relationship between interacting niche axes and microbial coexistence.

### Isolate selection

The bacterial strains used in these experiments were previously isolated from the fluid housed in 10 healthy pitchers of *S. purpurea* carnivorous pitcher plants at Harvard Pond (Petersham, MA) in September 2017 (see [46] for more sampling details). For these experiments, we selected the 26 isolate strains from 17 distinct families: *Alcaligenaceae, Alloboseaceae, Burkholderiaceae, Caulobacteraceae, Chitinophagaceae, Chromobacteriaceae, Comamonadaceae, Enterobacteriaceae, Microbacteriaceae, Oxalobacteriaceae, Pseudomonadaceae, Rhizobiaceae, Rhodanobacteraceae, Sphingobacteriaceae, Sphingomonadaceae, Weeksellaceae*, and * Yersiniaceae* (Supplemental Table 1). Previous research confirmed that all 26 isolates grew in cricket media, which was formulated by adding 3 g of food-grade cricket powder from farmed *Acheta domestica* crickets (Thailand Unique) to 1000 ml of distilled water [46]. Growth curves in Reasoner’s 2A (R2A) and M9 + casamino acids (CAAs) media (with and without added glucose) were obtained for each isolate by inoculating cells in sterile media and measuring optical density at 600 nm (OD_600_) every 5 min for 48 h (R2A) or 96 h (M9; longer time was required to accommodate for slower growth) on a SynergyMX plate reader. Growth rates were calculated in R using the Growthcurver v0.3.1 package (Supplemental Fig. 1). While all 26 selected strains grew in the R2A media, only 7 strains grew in simple M9 media amended with only glucose and salts. However, 10 strains grew once thiamine was added, and 25 of the strains grew after a final amendment with casamino acids. Even with these amendments, strain S33 belonging to the *Beijerinckiaceae* family showed no growth in M9 media. The 1X M9 media contained a final concentration of each of the following: 20 mM D-(+)-Glucose monohydrate (Thermo Scientific Cat. No. 047263.36), 0.1 mM CaCl_2_ (Alfa Aesar CAS: 10043-52-4), 2 mM MgSO_4_ (Thermo Scientific Cat. J61030.AK), 0.5 ug/ml thiamine HCL (Thermo Scientific Cat. 148990100), and 2% casamino acids (Teknova Cat. No. C2000). The R2A media were composed of 3.12 g of R2A broth (HiMedia M1687) in 1000 ml distilled water.

### Constructing and sampling synthetic bacterial communities

Prior to assembling the synthetic communities, isolate strains were grown from cryogenic stocks stored at −80°C onto R2A agar plates and allowed to grow until individual colonies were visible. Single colonies from each isolate plate were picked and used to inoculate 5 ml of sterile R2A or M9 minimal media liquid broth and cultures were incubated at room temperature with gentle shaking (300 rpm) for 6 days. After 6 days of growth, isolate cultures were standardized to approximately equivalent cell densities by diluting liquid cultures with sterile media to the lowest recorded isolate OD_600_ value (ranging from 0.20 to 0.38). Dilutions were verified with additional OD_600_ measures (BioTek SynergyMX plate reader) prior to assembling the synthetic communities. Synthetic communities were then assembled by combining equivalent volumes of each standardized isolate culture into a single mixed community.

Experiment 1 (25 strains, M9 vs. cricket media) and Experiment 2 (26 strains, R2A vs cricket media) were comparable but independent experiments. Each synthetic community was diluted at a 1:5 ratio using sterile media corresponding to the respective experimental treatments (R2A, M9, or cricket media). Once diluted, triplicate 1.5 ml aliquots of these experimental cultures were added to 96-well deep well plates and assigned to each of the treatments. Cultures were well mixed by pipetting and transferred into fresh sterile media at a 1:5 dilution every 3 days for a total of 12 transfers (39 days). At each transfer, 1 ml of culture from each sample was collected and frozen at −80°C for DNA extraction and downstream community composition analyses.

### Experimental treatments

To characterize the environmental axes of coexistence in bacterial communities, the synthetic bacterial communities were exposed to four distinct treatments in a full-factorial design, leading to 16 combinations (Fig. 1). The treatments were selected to investigate the relationship between multispecies coexistence and environmental structure, fluctuations, and resource complexity.

#### Spatial structure

Physical niche complexity was modified through two experimental conditions intended to alter the different physical spaces available within each treatment. Synthetic communities were exposed to (i) shaking vs. nonshaking conditions and (ii) the presence or absence of glass beads. Shaking samples were exposed to continuous gentle shaking (300 rpm) conditions, while nonshaking samples were kept in static conditions, which allowed for more stratification, increasing potential spatial niche complexity. Additional physical complexity was created by adding 1/32 of a teaspoon (~0.16 g) of sterile 0.5 mm glass beads (Biospec Cat. No. 11079105) to samples assigned to the “bead” condition, introducing small spatial pockets that allow for microbial attachment.

#### Temporal fluctuation

To explore the role of temporal fluctuations on multispecies coexistence, synthetic communities were assigned to either constant or fluctuating ambient air temperatures. Samples assigned to consistent temperatures were kept at room temperature, which ranged from 20°C to 22°C, whereas samples assigned to variable temperatures were exposed to temperatures fluctuating from 21°C to 30°C on 6-h cycles in an incubator (Thermo Scientific Heratherm) for the duration of each experiment (Supplemental Fig. 2). Exposure to variable temperatures was intended to increase temporal fluctuations and promote coexistence through the generation of temporal niches for taxa with differing thermal preferences. Although we designed these temperature conditions to investigate the influence of temporal niches, we acknowledge that responses to the fluctuating temperature conditions could also be due to the exposure to higher temperatures rather than a fluctuating environment.

#### Resource complexity

Three distinct types of bacterial media were utilized to represent a range of resource complexity. M9 minimal medium (Difco 248510), amended with CAAs, salts, thiamine, and glucose, served as the lowest complexity treatment. In this formulation, casamino acids functioned as the primary carbon source, while glucose contributed some additional organic carbon. In contrast, our intermediate complexity media, R2A, was a low-nutrient medium designed for microbes growing in tap water but is an undefined medium with multiple carbon sources, including dextrose, yeast extract, casein acid hydrolysate, and starch. Finally, our cricket-based media contained several carbon sources in more complex forms, such as chitin, proteins, and fats, and therefore represented the highest resource complexity. This cricket media were not only a complex resource but also representative of the nutrient profiles typically available to the bacterial strains in their natural pitcher plant environments. Therefore, cricket media always represented the higher resource complexity condition. M9 medium was used as the lower complexity resource in the 25-strain experiment (Experiment 1), whereas the R2A medium was used as a lower complexity resource in the 26-strain experiment (Experiment 2).

### Custom reference taxonomy database

We used 16S rRNA gene Sanger sequencing of the 26 isolates to develop a custom taxonomic reference database. Microbial isolate DNA was obtained by adding 1–2 mm of sterile 100-micron low-binding zirconium beads (Ops Diagnostics NC1147014) to each isolate culture aliquot and bead beating (BioSpec Cat. No. 1001) samples at 2400 rpm for 5 min. An aliquot of each isolate culture was then removed and diluted at a 1:10 ratio with sterile, nuclease-free water. Diluted DNA samples were amplified using the 16S rRNA gene–specific primers 27F, 5′-AGAGTTTGATCCTGGCTCAG-3′ and 1492R, 5′-GGTTACCTTGTTACGACTT-3′. The PCR thermal profile was set to 98°C for 30 s, followed by 30 cycles of 98°C for 10 s, 60°C for 10 s, and 72°C for 45 s, and concluded with a final elongation step at 72°C for 5 min. Amplification was verified by 1% agarose gel electrophoresis, and amplified samples were purified using CleanNGS magnetic particle solution (CleanNA CNGS-0050). Amplified and cleaned samples were quantified, diluted to 2.8 ng/μl, and 25 pmol of the 27F primer was added to each sample. DNA Sanger sequencing of purified templates was performed by GeneWiz (Azenta Life Science, NJ, USA). Taxonomic assignments in our custom database were confirmed using the National Center for Biotechnology Information (NCBI) BLAST rRNA database prior to use in amplicon classification (Supplemental Table 1). Reference sequences used in the custom classifier were submitted to NCBI GenBank under PX01290-PX401315.

### Amplicon sequencing

To measure the composition of experimental synthetic communities, whole community DNA was obtained by adding 1–2 mm of sterile 100-micron low-binding zirconium beads (Ops Diagnostics NC1147014) to each collected culture aliquot and bead beating (BioSpec Cat. No. 1001) samples at 2400 rpm for 5 min. DNA from bead-beaten samples was then purified using CleanNGS magnetic particle solution (CleanNA CNGS-0050), and concentrations were obtained using an AccuClear Ultra High Sensitivity dsDNA Quantitation Assay (Biotium Cat. No. 50-196-4547) quantified using a BioTek SynergyMX plate reader. Duplicate negative controls composed of sterile media used in each experiment were also bead beaten and amplified to check for potential contamination prior to sequencing. We had previously verified that all our experimental strains could be amplified through simple colony PCR and that our DNA extraction approach was as effective as a kit-based extraction. Library preparation and Illumina amplicon sequencing of purified templates was performed by SeqCoast Genomics, LLC (Portsmouth, NH, USA). Briefly, DNA samples were amplified using Q5 High-Fidelity 2X Master Mix (NEB) with the 515F (GTGYCAGCMGCCGCGGTAA) and 806R (GGACTACNVGGGTWTCTAAT) primers targeting the V4 region of the 16S rRNA gene. Sequencing was performed on the NextSeq2000 System (Illumina) using a 300-cycle flow cell to produce 2 × 150 bp paired reads spiked with 30%–40% PhiX. Read demultiplexing, adapter trimming and run analytics were performed by SeqCoast using DRAGEN v4.2.7. The two experiments were sequenced on separate runs.

Additional processing of the demultiplexed amplicon sequences was performed with QIIME2 (2021.4). Using the DADA2 [49] module, forward and reverse primers were trimmed, sequences were denoised, merged and quality filtered, and amplicon sequence variants (ASVs) were generated. Taxonomy was assigned using the Naive Bayes Classifier (classify-sklearn) pre-trained on a custom reference database containing 16S rRNA gene sequences obtained from the bacterial isolates used to construct our synthetic communities. Taxonomic classifications were further verified and potential contaminant sequences were identified using the SILVA v138 database [50]. All strains were accurately classified and contaminant sequences were universally low (<1% of reads). For Experiment 1, 194 samples were processed yielding a total of 64 862 677 sequences and 6832 ASVs. Samples were then rarefied to 17 841 sequences and individual ASVs were binned according to their taxonomic assignment from our custom classifier. After processing and rarefying our sequences, we retained all 194 of our samples. For Experiment 2, 194 samples were processed yielding a total of 184 317 065 sequences and 5789 ASVs. Samples were then rarefied to 7313 sequences and individual ASVs were binned according to their taxonomic assignment from our custom classifier. After processing and rarefying our sequences, we retained all 194 of our samples. Rarefaction curves confirmed that the selected sampling depth captured the full taxonomic diversity (Supplemental Fig. 3). Taxa accounting for <1% of reads in any given sample were aggregated and reclassified as rare taxa. Across all samples, unassigned reads accounted for ~51% of our ASVs but only 0.25% of all sequence counts. The raw sequence files were submitted to NCBI under BioProject accession number PRJNA1333501.

### Phylogenetic tree

We constructed a phylogenetic tree to explore the evolutionary relationships among the 26 bacterial taxa based on the 16S rRNA gene. A sequence from Candidatus *Nitrosocosmicus* (an archeon) was obtained from NCBI GenBank [51] and was used as an outgroup reference to ensure that the tree was rooted with a phylogenetically distinct species. We aligned these sequences using MUSCLE and trimmed the overhangs to obtain a cleaner, more accurate phylogenetic analysis [52]. Using the Tree and reticulogram REConstruction (T-Rex) web server, we generated a RAxML maximum likelihood phylogenetic tree with default parameters and a bootstrap value of 500 [53]. The best tree was then visualized using the Interactive Tree of Life program (ITOL) [54].

### Genome sequencing and analysis

To obtain genome sequences for each of our strains, isolates were either grown from cryogenic stocks or single-plated colonies in 13 ml of liquid R2A. Isolate cultures were incubated at room temperature with gentle shaking (300 RPM) for 10 days. Cultures were then centrifuged at 10 000 RPM for 5 min, and the supernatant was carefully discarded while the bacterial cell pellets were left undisturbed. The cell pellets were then washed with 1 ml of 1× sterile Phosphate-Buffered Saline (PBS) and centrifuged a second time. After carefully discarding the supernatant, the bacterial cells were resuspended in 500 μl of Zymo 1× DNA/RNA Shield. Bacterial DNA extraction and genome sequencing was performed by Plasmidsaurus using Oxford Nanopore Technology with custom analysis and annotation. Briefly, bacterial cell pellets were sequenced with Oxford Nanopore (ONT) long reads with a primer-free protocol using R10.4.1 flow cells. Once sequenced, the bottom 5% lowest-quality reads were removed with Filtlong v0.2.1. Remaining sequences were assembled using Flye v2.9.1 and polished using Medaka v1.8.0. Assembled genomes were annotated using Bakta v1.6.1, and genome completeness and contamination were checked using CheckM v1.2.2. The final ONT genomes were then polished using Illumina shotgun sequences to generate high-quality hybrid assembled genomes. All genome assemblies were high quality (>97% complete) with minimal contamination (<3%, Supplemental Table 1). Assembled genomes were submitted to the NCBI under BioProject accession number PRJNA1337152.

### Data analysis

All statistical analyses were conducted in R version 4.5.1 [55]. To address our first hypothesis, we tested how diversity related to niche complexity. Niche complexity was quantified by assigning a value from 0 to 4 to each experimental treatment based on the number of complex conditions represented, such that the lowest-complexity conditions (S.M9.RT.NB and S.R2A.RT.NB) were assigned a score of 0 whereas the highest complexity condition (NS.CR.VT.B) was assigned a score of 4. Species diversity at the final time step of each replicate was quantified using Hill numbers, calculated with the *hill_taxa* function in the hillR package [56]. Although richness (Hill Number 0), effective number of species (ENS, commonly known as the exponent of Shannon diversity, Hill Number 1), and Inverse Simpson Index (Hill Number 2) were calculated for each experiment (Supplemental Fig. 4), our analyses focused primarily on the effective number of species (ENS), which accounts for both species richness and evenness and is thus the most relevant metric of diversity for our study. The ENS at our final sampling point quantifies the number of strains that are well represented in the community after ~60 generations; however, we recognize that it is only an approximation of coexistence and would need to be explicitly tested in future studies [7]. The relationships between diversity (ENS) and overall treatment complexity were evaluated using mixed-effects models to account for the nonindependence of treatments arising from the fully factorial design. Specifically, we fit linear, quadratic, and cubic models with treatment identity included as a random effect. Environmental complexity was modeled as a continuous predictor, and model fits were compared using Akaike Information Criterion and marginal *R*^2^ values. All inferential analyses examining the roles of each treatment and their interactive effects were conducted using fully factorial models that explicitly included individual environmental factors and potential interactions.

To test whether the observed saturation in diversity could be explained by sampling constraints imposed by a finite species pool, we constructed a null model based on the experimentally relevant starting communities. For each experiment, we first defined the initial species pool using Day 0 samples. Relative abundances for each taxon were averaged across the replicate initial communities within each experiment to obtain a single pooled species abundance distribution. We then generated null communities by randomly sampling individuals from this pooled distribution. For each experiment, we performed 1000 independent simulations in which 100 individuals were drawn with replacement such that relative and absolute abundance were equal, with sampling probabilities proportional to each species’ mean initial relative abundance. This approach preserves both the identity and relative abundance structure of the initial species pool while removing any effects of ecological interactions during community assembly. For each simulated community, relative abundances were calculated from the sampled counts, and diversity was quantified using the effective number of species (ENS; Hill number with *q* = 1) using the hillR package, mirroring our data processing workflow for observed diversity measures. This produced a distribution of expected ENS values under a purely sampling-driven assembly process. Observed ENS values from experimental communities were then compared to this null distribution to assess whether diversity patterns could be explained by sampling from the finite initial species pool alone.

To address our second hypothesis, the relationships between final ENS values and individual treatment conditions were investigated with generalized linear models (GLMs) using a gamma distribution with a log link, implemented with the *glm* function in R. GLMs were run on each experiment independently with full interactive effects (formula = ENS ~ Media*Shake*Temperature*Beads). The lowest-complexity treatment in each experiment (S.M9.RT.NB and S.R2A.RT.NB) was used as the reference level in their respective GLMs. Under a log-link GLM, effects combine multiplicatively such that interaction terms represent deviations from the expected product of individual factor effects. For all GLM models, results were visualized using the *ggpredict* function from the ggeffects package v2.3.0 [57] and plotted using ggplot2 package v3.5.2 [58]. Community trajectory and stability were analyzed using beta diversity calculated as Bray–Curtis dissimilarity values using the *metaMDS* function in the vegan package v2.7 [59]. Community composition was compared across treatments using permutational multivariate analysis of variance (PERMANOVA) implemented with the *adonis2* function in the vegan package v2.7, using Bray–Curtis dissimilarity matrices and 999 permutations. Treatment was included as a categorical predictor representing each unique combination of environmental conditions, allowing us to assess overall differences in community composition among experimental treatments. To evaluate the consistency of community assembly within treatments, we tested for homogeneity of multivariate dispersion using the *betadisper* function followed by ANOVA in R.

To investigate potential biotic drivers of coexistence within the system, strain-level responses to our experimental conditions were identified by performing differential abundance analysis using ANCOM-BC2 [60] with full interactive effects (formula = ~Media^*^Shake^*^Temperature^*^Beads) and reference levels set to the lowest complexity treatment in each experiment. Raw sequence counts were used as input for ANCOM-BC2, which accounts for compositionality and differences in sampling depth through an internal bias-correction framework, eliminating the need for prior normalization or transformation. R code, input data, and additional analysis details are available in our public repository: github.com/jgronniger/Microbial-Coexistence.

## Results and discussion

### Environmental complexity shapes diversity through interacting niche axes

**Figure 2:**
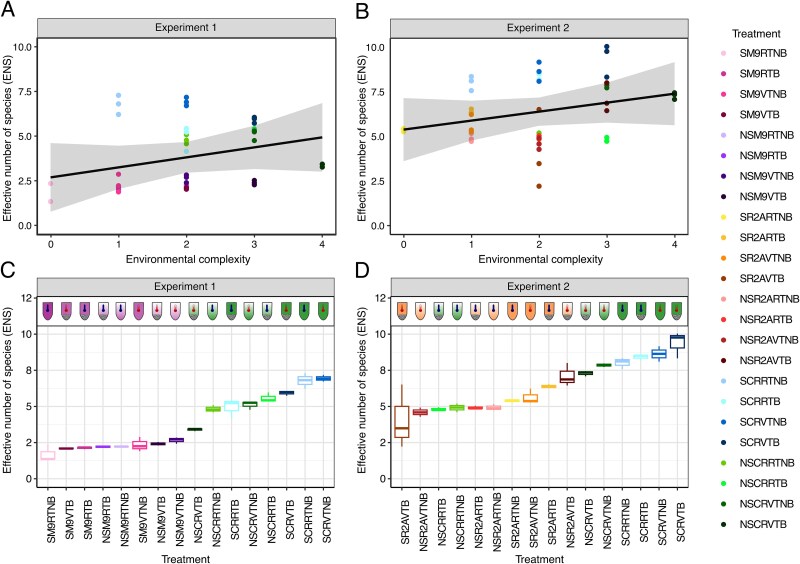
Relationship between effective number of species (ENS) and environmental complexity at final timepoint (Day 39). The relationship between ENS and overall environmental complexity was investigated by assigning a complexity value to each treatment based on the number of complex conditions included, such that the lowest-complexity treatments, S.M9.RT.NB and S.R2A.RT.NB, are assigned a score of 0, whereas the highest complexity treatment, NS.CR.VT.B, was assigned a score of 4. Points represent individual communities colored by treatment. Solid lines show the fitted relationship from the best-supported mixed-effects model (linear in both experiments), with treatment included as a random effect to account for nonindependence among conditions. Marginal *R*^2^ values were 0.09 (*P* = .206) in (A) Experiment 1 and 0.08 (*P* = .218) in (B) Experiment 2. Boxplots show the ENS for each treatment condition in (C) Experiment 1 and (D) Experiment 2, ordered by ascending ENS values. Treatments are indicated by both abbreviations and icons representing their respective environmental conditions.

To address our first hypothesis of a positive relationship between niche complexity and the maintenance of diversity, we compared the ENS at our final timepoint (Day 39, after 12 transfers) across each of the experimental treatments. Although we hypothesized a positive relationship between environmental complexity and diversity, the observed trend in both experiments was not statistically significant when controlling for treatment identity (Fig. 2A and B). These results indicated that niche complexity alone did not have an additive effect; instead, changes in diversity were context-dependent and varied across media and experimental conditions. Although estimated marginal means suggested a slight increase at intermediate complexity levels, sequential contrasts between adjacent levels of complexity (0 → 1, 1 → 2, 2 → 3, 3 → 4) were small and not significantly different from zero (all *P* > .05), indicating no consistent monotonic relationship. In both experiments, the treatment with the highest complexity score of 4 did not have the highest ENS, showing that combining higher levels of environmental complexity will not necessarily lead to the maintenance of higher diversity (Fig. 2A and B).

To determine whether saturation in diversity could be explained by sampling constraints imposed by the finite initial species pool, we constructed a null model based on the starting community composition (Supplemental Fig. 5). This analysis showed that observed ENS values remained well below the diversity expected under random sampling from the initial pool, indicating that the saturating pattern is not driven by species pool limitation but instead reflects ecological constraints on coexistence. Such constraints have been documented within individual niche axes, particularly in relation to resource complexity [61, 62], and, to a more limited extent, across axes in plant systems [63]; but to our knowledge, have not been demonstrated across interacting niche dimensions in microbial systems as observed here.

When looking at patterns leading to higher ENS in our treatments (Fig. 2C and D), the influence of media type was particularly apparent in Experiment 1, where treatments containing the complex cricket media consistently had higher ENS values than treatments containing the simpler M9 + CAA + glucose media regardless of the other treatment conditions (Fig. 2C). Experiment 2 displayed a similar, yet less stringent, pattern, where six of the treatments with the highest ENS all contained the complex cricket media (Fig. 2D). However, two of the cricket media treatments in Experiment 2 (NS.CR.RT.B and NS.CR.RT.NB) had notably lower ENS values than other comparable cricket treatments in the experiment, suggesting that interactions between media type and additional environmental conditions were likely present [64, 65].

We used the ENS, the Hill number with *q* = 1, because it incorporates both species richness and evenness while reducing the influence of rarer species, providing a measure that emphasizes dominant species that persisted at the final time points. To assess the robustness of these patterns, we also calculated diversity using Hill numbers with *q* = 0 (richness) and *q* = 2 (greater weighting of dominant species). These alternative metrics yielded similar patterns (Supplemental Fig. 4), indicating that our conclusions are consistent across diversity measures. After 12 transfers, community composition showed minimal temporal change, suggesting likely stabilization of community dynamics (Supplemental Fig. 6). However, determination of true equilibrium coexistence would require testing for mutual invasibility or other metrics of coexistence across of all taxa [8].

### Different axes of niche space shape diversity through interactive effects

To address our second hypothesis that different axes of niche space would work synergistically to support increased diversity, we ran fully interactive GLMs for each of our experiments. Using the respective low-complexity treatments as reference levels (S.R2A.RT.NB and S.M9.RT.NB), we quantified the relative independent and interactive effects for each of our four treatment conditions on our final ENS values.

**Figure 3:**
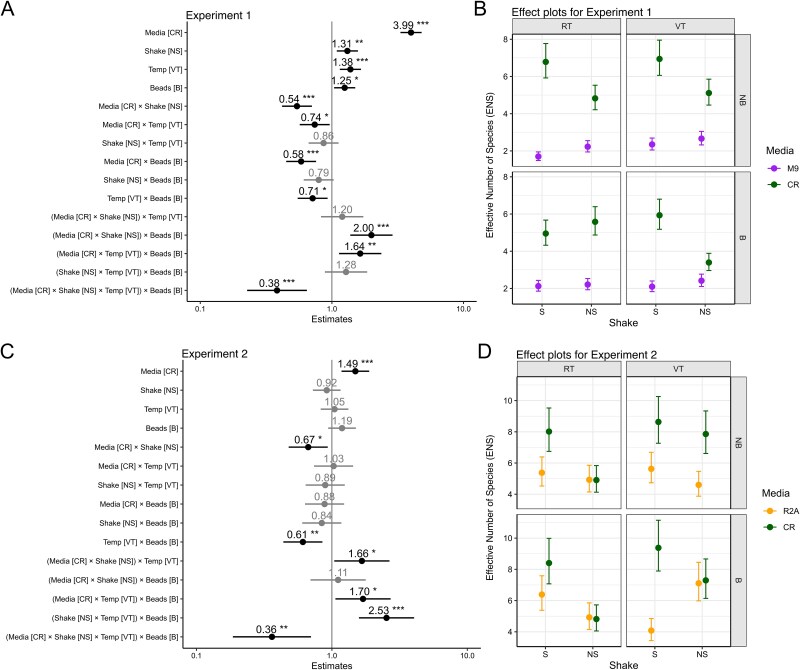
Generalized linear model (GLM) results showing the independent and interactive effects of shaking, media type, temperature, and bead treatments on the effective number of species (ENS) at the final timepoint (Day 39) for (A) Experiment 1 and (C) Experiment 2. The models were fitted using a gamma distribution with a log link function to account for the skewed distribution of the positive response variable. The lowest complexity treatment (S.M9.RT.NB in A and S.R2A.RT.NB in C) was used as our reference level. The generalized linear models explained 96% and 82% of variance in species diversity for Experiments 1 and 2, respectively. Estimated model coefficients and 95% confidence intervals are displayed on the exponentiated (effect size) scale. Statistically significant effects are labeled with asterisks indicating the level of significance (`^*^` *P* < .05, `^**^` *P* < .01, `^***^` *P* < .001). Non-significant values are shown in gray. The vertical black line indicates the null value. Model estimates for (B) Experiment 1 and (D) Experiment 2 are shown as interaction effects plots illustrating the combined influence of the four-way interaction between shaking, media, temperature, and beads on ENS values. Predicted estimates and 95% confidence intervals across levels of interacting predictors are displayed and points are colored by media type. Treatment factors are abbreviated as follows: S, shaking, NS, no shaking; M9 = M9 + CAA + glucose; CR, cricket media, RT, constant room temperature; VT, variable temperature; B, beads present; NB, no beads.

Results showed strong independent and interactive effects (Fig. 3). Because several interaction terms were statistically significant, we focused interpretation on these first. Across both experiments, significant pairwise interactions were consistently lower than 1, indicating that combined effects were often weaker than expected under multiplicative independence. For example, the interaction coefficients for Media × Shake and Temperature × Beads were reduced in both Experiment 1 (≈ 0.54 and ≈ 0.71, respectively) and Experiment 2 (≈ 0.67 and ≈ 0.61), demonstrating attenuation across certain conditions. Conversely, most of the three-way interactions were >1 (e.g. Media × Shake × Beads ≈ 2.00 in Experiment 1 and ≈ 1.66 in Experiment 2), indicating frequent context-dependent synergy. Notably, the four-way interaction was significantly <1 in both experiments (≈ 0.38 and ≈ 0.36), consistent with saturation at the highest level of complexity.

We interpreted the main effects in the context of the interactive effects. Of the main effects, media composition had the strongest positive effect in both Experiment 1 (≈ 3.99) and Experiment 2 (≈ 1.49). In both experiments, none of the higher-complexity conditions had negative independent effects on diversity, and in Experiment 1, all were significantly positive (Fig. 3). However, these main effects were clearly modulated by interactions with other factors. For example, the combined effect of cricket media and no shaking led to lower-than-expected ENS values in both experiments. Whereas we originally postulated that the no-shaking condition would lead to higher diversity by increasing spatial niche availability through stratification, model estimates suggest that this was not always the result. Perhaps, under certain conditions, a decrease in oxygen due to no shaking led to decreased survival and diversity [66, 67]. Together, these results indicate that the effects of niche complexity are nonmultiplicative and context dependent, with limited evidence for consistent synergy and clear evidence of attenuation at higher levels of complexity (Fig. 3).

### Community composition over time and across treatments

To elucidate potential community-level drivers of our observed diversity–complexity relationship, we first investigated how community composition changed across our treatments and over time. We visualized overall shifts in community composition using nonmetric multidimensional scaling (NMDS) ordination based on Bray–Curtis dissimilarities at Day 0 (initial inoculum), 3 (3 days after experimental incubation), and 39 (final timepoint) for Experiments 1 and 2 (Supplemental Fig. 7). NMDS ordination revealed clear shifts in community composition over time and in response to experimental treatments. Clustering of communities at Day 0 in both plots confirm that our initial synthetic communities shared similar compositions as intended. Similarities in starting inocula were further confirmed using composition plots (Supplemental Fig. 8). However, after only 3 days of exposure to experimental treatments, community compositions had shifted from the starting inocula regardless of treatment conditions, indicating that the synthetic communities exhibited rapid changes upon incubation, consistent with other synthetic community incubations [68, 69]. By day 39, pronounced separation emerged across most treatments, particularly among Experiment 1 samples that displayed a notable split between media types (Supplemental Fig. 7A).

**Figure 4:**
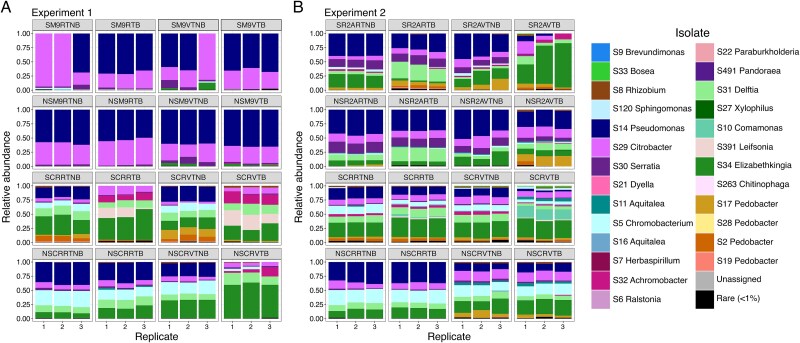
Final community composition across treatments and replicates. (A, B) Stacked bar plots showing community composition across all three replicates on Day 39, faceted by treatment conditions for (A) Experiment 1 and (B) Experiment 2. Bar plots are arranged according to our experimental design in Fig. 1. Colors correspond to individual isolates, labeled by strain number and corresponding genus. Full taxonomic classifications are available in Supplemental Table 1. Isolates with no taxonomic assignment are displayed in light gray. Isolates with a relative abundance <1% in any given sample were grouped into a rare category, displayed in black.

The strong differences in community composition across treatments were replicable, as illustrated by the overall consistency across our three replicates in both experiments (Fig. 4C and 4D). Communities in M9 media were by far the simplest, being dominated by two taxa: a *Pseudomonas* species (S14) and a *Citrobacter* species (S29; Fig. 4C). The consistent prevalence of these two taxa across all M9 treatments further supports our observations that media type plays a significant role in shaping these synthetic communities. As a media with relatively simple carbon sources (CAA and glucose), microbes grown in M9 likely experience strong competitive interactions modulated by growth rate, metabolic capacities, and biotic interactions [35, 65, 70]. For example, strains have been shown to successfully coexist on limited simple resources through the partitioning of carbon sources where respirofermenters like *Citrobacter* consume the sugars first and secrete organic acids that can then be consumed by respirers like *Pseudomonas*, effectively supporting stable coexistence through resource partitioning and cross-feeding [71]. It is likely that this coexistence through cross-feeding between the *Pseudomonas* and *Citrobacter* species is a common dynamic as both strains persist in most of our treatments. However, exposure to increasingly complex media appears to modulate these biotic interactions, likely through a combination of increased resource complexity and availability leading to more complex cross-feeding interactions and subsequent niche space creation [28, 29]. Future research could quantify how the strengths of potential species interactions change across a gradient of niche complexity in order to elucidate how modified biotic interactions contribute to coexistence dynamics.

Final community composition differed significantly among treatments in both experiments (PERMANOVA, Experiment 1: *R*^2^ = 0.90, *P* = .001; Experiment 2: *R*^2^ = 0.92, *P* = .001), indicating strong treatment-level structuring of microbial communities. Tests of multivariate dispersion showed no significant differences in within-treatment variability in either experiment (Experiment 1: *P* = .564; Experiment 2: *P* = .091), indicating that the PERMANOVA results are trustworthy. Furthermore, in both experiments, mean within-treatment dissimilarity was significantly lower than between-treatment dissimilarity (Wilcoxon tests, *P* < 2.2 × 10^−16^), indicating that communities were more similar within treatments than across treatments. These results demonstrate that environmental conditions strongly structure community composition and lead to repeatable assembly outcomes [70]. For example, two treatments (NS.CR.RT.NB and NS.CR.RT.B) produced nearly identical final compositions across both experiments despite being conducted independently (Fig. 4), suggesting that certain treatments exert strong deterministic effects. However, all communities grown in M9 under no-shaking conditions converged on similar final compositions regardless of bead or temperature treatments (Fig. 4A), indicating that some environmental conditions impose stronger constraints on community assembly than others, leading to unequal contributions of different niche axes to coexistence [63]. Together, these results indicate that environmental conditions strongly constrain community assembly, leading to consistent outcomes within treatments, while still allowing for context-dependent variation in taxonomic composition.

**Figure 5:**
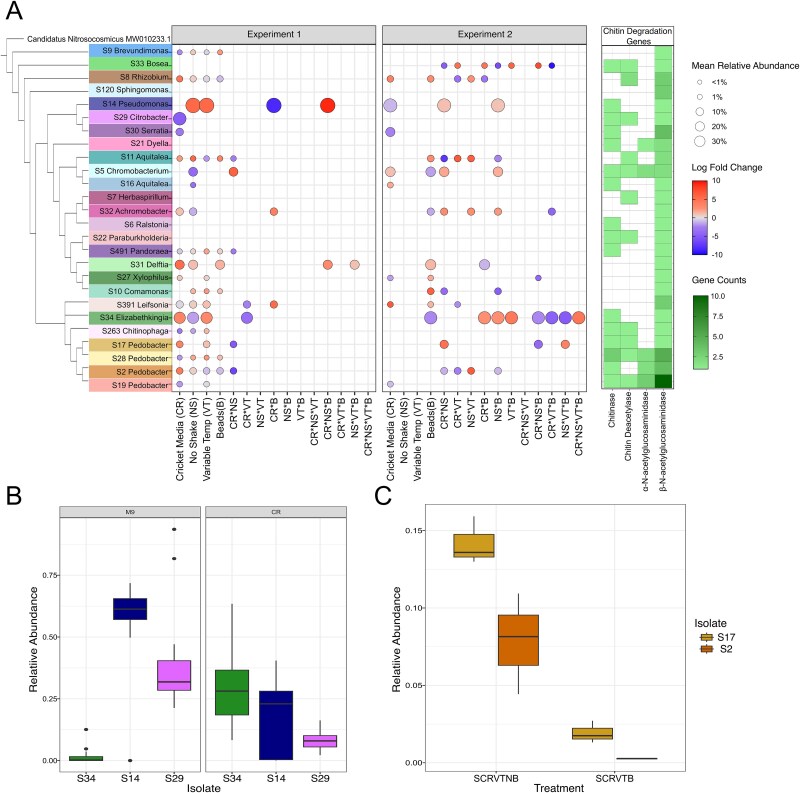
(A) Strain-level responses to treatment factors at final timepoint (Day 39) displayed as log fold change values calculated using a fully interactive formula (Media^*^Shake^*^Temp^*^Beads) in ANCOM-BC2. Strains are displayed on the left, ordered by phylogeny. Significant log fold change values are indicated by color, and average relative abundance of each strain is indicated by the size of each dot. Experiments were analyzed independently using the least complex treatment in Experiment 1 (S.M9.RT.NB) and Experiment 2 (S.R2A.RT.NB) as reference levels. Counts of genes related to chitin degradation obtained from each isolate genome are displayed in the right-hand heatmap. Comparison of relative abundances at final timepoint (Day 39) of Experiment 1 for (B) three strains of interest (S34, S14, and S29) across all treatments faceted by media type and (C) for the two *Pedobacter* strains (S17 and S2) in treatments S.CR.VT.NB and S.CR.VT.B.

### Individual species responded to the different environmental conditions

To explore potential mechanisms underlying the community-level patterns described above, we investigated strain-level responses to individual and interacting environmental factors using differential abundance analysis on final experimental communities (Fig. 5). Differential abundance analyses for microbial relative abundance data have known limitations [72]; thus, we chose to use ANCOM-BC2 as it represents one of the most robust available approaches, particularly due to its improved control of false discovery rates and reduced compositional bias [73]. This analysis was not intended to simply demonstrate compositional differences among treatments, which are already evident at the community level, but to assess whether strain responses are predictable across contexts or instead contingent on specific combinations of environmental factors. Whereas most strains were found to be differentially abundant in at least one environmental condition, these differences were not consistent within phylogenetic clusters nor between experiments, suggesting context-dependent strain-level responses, likely both to environmental conditions and shifting species interactions [74]. For example, the prominent *Pseudomonas* species (S14) exhibited a strong positive differential abundance in no shaking and variable temperature conditions in Experiment 1 but had no significant differential abundance with individual factors in Experiment 2, although we noted positive differential abundance in the interactive conditions of no shaking with cricket media or beads (Fig. 5A). This inconsistency highlights that strain responses cannot be inferred from single environmental factors alone but instead depend on interactive effects between treatments [75, 76]. In contrast, the *Serratia* species (S30) was the only strain with a consistent response across both experiments, showing higher differential abundances in the simpler media types. Overall, these patterns indicate that strain-level responses are highly context-dependent and do not follow simple, generalizable rules based on phylogeny or individual environmental axes [7, 70]. We noted that the five strains that consistently dominated the NS.CR.RT.B and NS.CR.RT.NB conditions (S14, S29, S5, S31, S34) did not exhibit consistent differential abundance patterns across environmental factors despite resulting in nearly identical final compositions in both experiments. This decoupling between differential abundances and final community composition further supports the idea that community assembly outcomes emerge from interactions among strains rather than independent responses to environmental variables [7]. Three of these strains had chitinase genes within their genomes (Fig. 5A), but only one, an *Elizabethkingia* species (S34), had a positive differential abundance in cricket media, whereas the other two, the *Pseudomonas* (S14) and *Citrobacter* species (S29) had negative differential abundances in cricket media (Fig. 5B). This mismatch between genomic potential and abundance responses suggests that functional traits alone are insufficient to predict outcomes without considering ecological context [65, 77]. From past experiments [46], we know that the *Elizabethkingia* strain S34 has high protease activity whereas S14 and S29 do not. This suggests that S34 may gain a competitive advantage by being a primary degrader of cricket proteins, whereas S14 and S29 conduct secondary degradation and cross-feeding, effectively promoting coexistence through resource partitioning [28, 29]. This pattern is consistent with a mechanism in which primary degraders facilitate coexistence by generating secondary resources that support additional taxa, rather than all strains responding independently to the same resource pool [64, 78].

Beyond media types, we also assessed how additional environmental factors modulated the ways in which strains within a community responded. For example, in Experiment 1, two *Pedobacter* species (S2 and S17) exhibited very different relative abundances in treatments differing only the in presence of glass beads, representing ~0% and 2% of the community, respectively, in S.CR.VT.B and ~8% and 14%, respectively, in S.CR.VT.NB (Fig. 5C). Although the genomes of both *Pedobacter* species contain chitinase genes and both had positive differential abundances in cricket media, it appears that the two species were only able to benefit from the cricket media in the absence of beads. This suggests that the presence of beads may have a negative effect on their ability to utilize chitin as a resource either by acting as a mechanical disturbance (lysing sensitive cells) or by providing additional niche space that preferentially benefits strains that could then outcompete the *Pedobacter* strains in the presence of beads. This provides an example of how physical structure interacts with resource availability to shape strain-level outcomes, likely by altering competitive dynamics or access to resources.

## Conclusions

We did not observe a significant positive relationship between diversity and the number of higher environmental complexity levels when controlling for treatment identity, suggesting that simply combining different axes of niche variation will not necessarily lead to the long-term retention of more species within our synthetic communities (Fig. 2). Instead, the effects of resource complexity depended on the environmental context, as interactions with spatial and temporal variation produced both synergistic and antagonistic outcomes. Across two independent experiments, resource complexity consistently emerged as a primary driver of diversity while also exhibiting interactive effects with other niche axes (Fig. 3). The combination of all our more-complex conditions led to lower-than-expected species diversity, suggesting that added niche dimensionality may lead to saturating effects on species richness, with the potential for negative impacts. At the strain level, we found that responses to environmental complexity were highly context-dependent and could not be predicted from individual factors or phylogenetic relatedness alone. Despite these strong context dependencies, these analyses offer insight into how interactions among environmental axes translate into community-level outcomes. The strain-level data are consistent with the idea that environmental context reshapes interaction networks among strains, likely through changes in resource use and cross-feeding dynamics within these communities. It is likely that nonlinear diversity patterns emerge from shifts in strain-level interactions, including resource partitioning, facilitation, and competition, which vary across environmental contexts. Collectively, these results highlight that increasing niche dimensionality can aid in the maintenance of increased diversity but that these effects are contingent on interactions among niche axes and may diminish or saturate at higher levels of complexity. Future research should aim to further elucidate this contextual relationship by testing interactions between additional axes of niche complexity across systems, as well as other purported mechanisms of coexistence, such as fluctuating interaction strengths, metabolic switching, and/or partitioning and eco-evolutionary dynamics and trophic interactions.

## Supplementary Material

MXC_Supplemental_Tables_and_Figures_v3_wrag144

## Data Availability

Datasets generated and subsequent analyses for the current study are available in the Zenodo repository doi:10.5281/zenodo.20492567.

## References

[1] Clark JS, Dietze M, Chakraborty S., et al. Resolving the biodiversity paradox. *Ecol Lett* 2007;10:647–59. 10.1111/j.1461-0248.2007.01041.x 17594418

[2] Hutchinson GE . The paradox of the plankton. *Am Nat* 1961;95:137–45. 10.1086/282171

[3] Macarthur R, Levins R. The limiting similarity, convergence, and divergence of coexisting species. *Am Nat* 1967;101:377–85. 10.1086/282505

[4] Collings JA, Shoemaker LG, Diez JM. Environmental context alters plant–soil feedback effects on plant coexistence. *Ecology* 2025;106:e70170. 10.1002/ecy.70170 40767448 PMC12327179

[5] Molina-Venegas R, Verdú M, Montesinos-Navarro A., et al. Indirect reciprocal facilitation promotes species coexistence in plant communities worldwide. *Nat Ecol Evol* 2025;9:1373–81. 10.1038/s41559-025-02766-9 40555801

[6] Si M, Wang Z, Liu Y., et al. Individual asymmetric competition responses across multidimensional niches may enable coexistence of closely related species. *Funct Ecol* 2025;39:1957–71. 10.1111/1365-2435.70088

[7] Chang CY, Bajić D, Vila JCC., et al. Emergent coexistence in multispecies microbial communities. *Science* 2023;381:343–8. 10.1126/science.adg0727 37471535

[8] Chesson P . Mechanisms of maintenance of species diversity. *Annu Rev Ecol Syst* 2000;31:343–66. 10.1146/annurev.ecolsys.31.1.343

[9] Adler PB, Hillerislambers J, Levine JM. A niche for neutrality. *Ecol Lett* 2007;10:95–104. 10.1111/j.1461-0248.2006.00996.x 17257097

[10] Harmáčková L, Remešová E, Remeš V. Specialization and niche overlap across spatial scales: revealing ecological factors shaping species richness and coexistence in Australian songbirds. *J Anim Ecol* 2019;88:1766–76. 10.1111/1365-2656.13073 31329280

[11] McKane RB, Johnson LC, Shaver GR., et al. Resource-based niches provide a basis for plant species diversity and dominance in arctic tundra. *Nature* 2002;415:68–71. 10.1038/415068a 11780117

[12] Grotta-Neto F, Vogliotti A, de OML., et al. Brocket deer niche breadth and overlap: spatial similarities limit species coexistence. *J Zool* 2024;324:21–33. 10.1111/jzo.13180

[13] Levine JM, HilleRisLambers J. The importance of niches for the maintenance of species diversity. *Nature* 2009;461:254–7. 10.1038/nature08251 19675568

[14] Van Dyke MN, Levine JM, Kraft NJB. Small rainfall changes drive substantial changes in plant coexistence. *Nature* 2022;611:507–11. 10.1038/s41586-022-05391-9 36323782

[15] Hallett LM, Shoemaker LG, White CT., et al. Rainfall variability maintains grass-forb species coexistence. *Ecol Lett* 2019;22:1658–67. 10.1111/ele.13341 31298471

[16] Fierer N, Lennon JT. The generation and maintenance of diversity in microbial communities. *Am J Bot* 2011;98:439–48. 10.3732/ajb.1000498 21613137

[17] Brader G, Compant S, Mitter B., et al. Metabolic potential of endophytic bacteria. *Curr Opin Biotechnol* 2014;27:30–7. 10.1016/j.copbio.2013.09.012 24863894 PMC4045207

[18] Chiu HC, Levy R, Borenstein E. Emergent biosynthetic capacity in simple microbial communities. *PLoS Comput Biol* 2014;10:e1003695. 10.1371/journal.pcbi.1003695 24992662 PMC4084645

[19] Keasling JD . Manufacturing molecules through metabolic engineering. *Science* 2010;330:1355–8. 10.1126/science.1193990 21127247

[20] Allison SD, Martiny JBH. Resistance, resilience, and redundancy in microbial communities. *PNAS* 2008;105:11512–9. 10.1073/pnas.0801925105 18695234 PMC2556421

[21] McFall-Ngai M, Hadfield MG, Bosch TCG., et al. Animals in a bacterial world, a new imperative for the life sciences. *Proc Natl Acad Sci USA* 2013;110:3229–36. 10.1073/pnas.1218525110 23391737 PMC3587249

[22] Bittleston LS . Connecting microbial community assembly and function. *Curr Opin Microbiol* 2024;80:102512. 10.1016/j.mib.2024.102512 39018765

[23] Kallus Y, Miller JH, Libby E. Paradoxes in leaky microbial trade. *Nat Commun* 2017;8:1361. 10.1038/s41467-017-01628-8 29118345 PMC5678203

[24] Kim HJ, Boedicker JQ, Choi JW., et al. Defined spatial structure stabilizes a synthetic multispecies bacterial community. *Proc Natl Acad Sci USA* 2008;105:18188–93. 10.1073/pnas.0807935105 19011107 PMC2587551

[25] Blasche S, Kim Y, Mars R., et al. Metabolic cooperation and spatiotemporal niche partitioning in a kefir microbial community. *Nat Microbiol* 2021;6:196–208. 10.1038/s41564-020-00816-5 33398099 PMC7610452

[26] Jones SE, Lennon JT. Dormancy contributes to the maintenance of microbial diversity. *Proc Natl Acad Sci USA* 2010;107:5881–6. 10.1073/pnas.0912765107 20231463 PMC2851880

[27] Lopes W, Amor DR, Gore J. Cooperative growth in microbial communities is a driver of multistability. *Nat Commun* 2024;15:4709. 10.1038/s41467-024-48521-9 38830891 PMC11148146

[28] Dal Bello M, Lee H, Goyal A., et al. Resource–diversity relationships in bacterial communities reflect the network structure of microbial metabolism. *Nat Ecol Evol* 2021;5:1424–34. 10.1038/s41559-021-01535-8 34413507

[29] Hoek TA, Axelrod K, Biancalani T., et al. Resource availability modulates the cooperative and competitive nature of a microbial cross-feeding mutualism. *PLoS Biol* 2016;14:e1002540. 10.1371/journal.pbio.1002540 27557335 PMC4996419

[30] Coker J, Zhalnina K, Marotz C., et al. A reproducible and tunable synthetic soil microbial community provides new insights into microbial ecology. *mSystems* 2022;7:e0095122–2. 10.1128/msystems.00951-22 36472419 PMC9765266

[31] Kang H, Xue Y, Cui Y., et al. Nutrient limitation mediates soil microbial community structure and stability in forest restoration. *Sci Total Environ* 2024;935:173266. 10.1016/j.scitotenv.2024.173266 38759924

[32] Wortel MT . Evolutionary coexistence in a fluctuating environment by specialization on resource level. *J Evol Biol* 2023;36:622–31. 10.1111/jeb.14158 36799532

[33] Butler S, O’Dwyer JP. Stability criteria for complex microbial communities. *Nat Commun* 2018;9:2970. 10.1038/s41467-018-05308-z 30061657 PMC6065391

[34] Freilich S, Zarecki R, Eilam O., et al. Competitive and cooperative metabolic interactions in bacterial communities. *Nat Commun* 2011;2:589. 10.1038/ncomms1597 22158444

[35] Estrela S, Vila JCC, Lu N., et al. Functional attractors in microbial community assembly. *Cell Syst* 2022;13:29–42.e7. 10.1016/j.cels.2021.09.011 34653368 PMC8800145

[36] Madi N, Vos M, Murall CL., et al. Does diversity beget diversity in microbiomes? *elife* 2020;9:e58999. 10.7554/eLife.58999 33215610 PMC7755399

[37] Pacciani-Mori L, Giometto A, Suweis S., et al. Dynamic metabolic adaptation can promote species coexistence in competitive microbial communities. *PLoS Comput Biol* 2020;16:e1007896. 10.1371/journal.pcbi.1007896 32379752 PMC7244184

[38] Yuan C, Chesson P. The relative importance of relative nonlinearity and the storage effect in the lottery model. *Theor Popul Biol* 2015;105:39–52. 10.1016/j.tpb.2015.08.001 26307205

[39] Kuang JJ, Chesson P. Interacting coexistence mechanisms in annual plant communities: frequency-dependent predation and the storage effect. *Theor Popul Biol* 2010;77:56–70. 10.1016/j.tpb.2009.11.002 19945475

[40] Werner CM, Hallett LM, Shoemaker LG. Fluctuation-dependent coexistence of stage-structured species. *Am Nat* 2025;205:327–41. 10.1086/733382 39965226

[41] Letten AD, Dhami MK, Ke PJ., et al. Species coexistence through simultaneous fluctuation-dependent mechanisms. *Proc Natl Acad Sci* 2018;115:6745–50. 10.1073/pnas.1801846115 29895689 PMC6042140

[42] Lobanov A, Dyckman S, Kurkjian H., et al. Spatial structure favors microbial coexistence except when slower mediator diffusion weakens interactions. *elife* 2023;12:e82504. 10.7554/eLife.82504 37350317 PMC10348751

[43] Fridman Y, Wang Z, Maslov S., et al. Fine-scale diversity of microbial communities due to satellite niches in boom and bust environments. *PLoS Comput Biol* 2022;18:e1010244. 10.1371/journal.pcbi.1010244 36574450 PMC9829172

[44] Mattei M, Arenas A. Exploring spatial segregation induced by competition avoidance as driving mechanism for emergent coexistence in microbial communities. *Phys Rev E* 2024;110:014404. 10.1103/PhysRevE.110.014404 39160961

[45] Brochet S, Quinn A, Mars RA., et al. Niche partitioning facilitates coexistence of closely related honey bee gut bacteria. *elife* 2021;10:e68583. 10.7554/eLife.68583 34279218 PMC8456714

[46] Bittleston LS, Gralka M, Leventhal GE., et al. Context-dependent dynamics lead to the assembly of functionally distinct microbial communities. *Nat Commun* 2020;11:1440. 10.1038/s41467-020-15169-0 32188849 PMC7080782

[47] Grothjan JJ, Young EB. Diverse microbial communities hosted by the model carnivorous pitcher plant Sarracenia purpurea: analysis of both bacterial and eukaryotic composition across distinct host plant populations. *PeerJ* 2019;7:e6392. 10.7717/peerj.6392 30805246 PMC6383556

[48] Heil JA, Wolock CJ, Pierce NE., et al. Sarracenia pitcher plant-associated microbial communities differ primarily by host species across a longitudinal gradient. *Environ Microbiol* 2022;24:3500–16. 10.1111/1462-2920.15993 35384233

[49] Callahan B, McMurdie P, Rosen M., et al. DADA2: high resolution sample inference from Illumina amplicon data. *Nat Methods* 2016;13:581–3. 10.1038/nmeth.3869 27214047 PMC4927377

[50] Quast C, Pruesse E, Yilmaz P., et al. The SILVA ribosomal RNA gene database project: improved data processing and web-based tools. *Nucleic Acids Res* 2013;41:D590–6. 10.1093/nar/gks1219 23193283 PMC3531112

[51] Candidatus Nitrosocosmicus exaquare clone ZSH-B1 16S ribosomal RNA gene, partial sequence . 2024. NCBI Nucleotide Database, 1905074192. http://www.ncbi.nlm.nih.gov/nuccore/MW010233.1 (18 Aug. 2025, date last accessed).

[52] Madeira F, Madhusoodanan N, Lee J., et al. The EMBL-EBI job dispatcher sequence analysis tools framework in 2024. *Nucleic Acids Res* 2024;52:W521–5. 10.1093/nar/gkae241 38597606 PMC11223882

[53] Boc A, Diallo AB, Makarenkov V. T-REX: a web server for inferring, validating and visualizing phylogenetic trees and networks. *Nucleic Acids Res* 2012;40:W573–9. 10.1093/nar/gks485 22675075 PMC3394261

[54] Letunic I, Bork P. Interactive tree of life (iTOL) v6: recent updates to the phylogenetic tree display and annotation tool. *Nucleic Acids Res* 2024;52:W78–82. 10.1093/nar/gkae268 38613393 PMC11223838

[55] R Core Team . R: The R Project for Statistical Computing. R Foundation for Statistical Computing. 2025. https://www.r-project.org/ (1 Aug. 2025, date last accessed).

[56] Chao A, Chiu CH, Jost L. Unifying species diversity, phylogenetic diversity, functional diversity, and related similarity and differentiation measures through hill numbers. *Annu Rev Ecol Evol Syst* 2014;45:297–324. 10.1146/annurev-ecolsys-120213-091540

[57] Lüdecke D . Ggeffects: tidy data frames of marginal effects from regression models. *J Open Source Softw* 2018;3:772. 10.21105/joss.00772

[58] H. Wickham ggplot2: Elegant Graphics for Data Analysis. 2016. https://ggplot2.tidyverse.org (1 Aug. 2025, date last accessed).

[59] J Oksanen, GL Simpson, FG Blanchet. et al. vegan: an R package for community ecologists. 2025. https://vegandevs.github.io/vegan/ (1 Aug. 2025, date last accessed).

[60] Lin H, Peddada SD. Multigroup analysis of compositions of microbiomes with covariate adjustments and repeated measures. *Nat Methods* 2024;21:83–91. 10.1038/s41592-023-02092-7 38158428 PMC10776411

[61] Eisenhauer N, Schulz W, Scheu S., et al. Niche dimensionality links biodiversity and invasibility of microbial communities. *Funct Ecol* 2013;27:282–8. 10.1111/j.1365-2435.2012.02060.x

[62] Harpole WS, Sullivan LL, Lind EM., et al. Addition of multiple limiting resources reduces grassland diversity. *Nature* 2016;537:93–6. 10.1038/nature19324 27556951

[63] Yan X, Diez J, Huang K., et al. Beyond resource limitation: an expanded test of the niche dimension hypothesis for multiple types of niche axes. *Oecologia* 2020;193:689–99. 10.1007/s00442-020-04713-w 32681295

[64] Pacheco AR, Osborne ML, Segrè D. Non-additive microbial community responses to environmental complexity. *Nat Commun* 2021;12:2365. 10.1038/s41467-021-22426-3 33888697 PMC8062479

[65] Estrela S, Sanchez-Gorostiaga A, Vila JC., et al. Nutrient dominance governs the assembly of microbial communities in mixed nutrient environments. *elife* 2021;10:e65948. 10.7554/eLife.65948 33877964 PMC8057819

[66] Junkins EN, McWhirter JB, McCall LI., et al. Environmental structure impacts microbial composition and secondary metabolism. *ISME Commun* 2022;2:15. 10.1038/s43705-022-00097-5 37938679 PMC9723690

[67] Wilbert SA, Newman DK. The contrasting roles of nitric oxide drive microbial community organization as a function of oxygen presence. *Curr Biol* 2022;32:5221–5234.e4. 10.1016/j.cub.2022.10.008 36306787 PMC9772256

[68] Kleyer H, Tecon R, Or D. Rapid shifts in bacterial community assembly under static and dynamic hydration conditions in porous media. *Appl Environ Microbiol* 2019;86:e02057–19. 10.1128/AEM.02057-19 31653789 PMC6912082

[69] Meroz N, Tovi N, Sorokin Y., et al. Community composition of microbial microcosms follows simple assembly rules at evolutionary timescales. *Nat Commun* 2021;12:2891. 10.1038/s41467-021-23247-0 33976223 PMC8113234

[70] Goldford JE, Lu N, Bajić D., et al. Emergent simplicity in microbial community assembly. *Science* 2018;361:469–74. 10.1126/science.aat1168 30072533 PMC6405290

[71] Sun X, Favier A, Folmar J., et al. Metabolic plasticity shapes microbial communities across a temperature gradient. *Am Nat* 2024;204:381–99. 10.1086/731997 39326062

[72] Nearing JT, Douglas GM, Hayes MG., et al. Microbiome differential abundance methods produce different results across 38 datasets. *Nat Commun* 2022;13:342. 10.1038/s41467-022-28034-z 35039521 PMC8763921

[73] Yang L, Chen J. A comprehensive evaluation of microbial differential abundance analysis methods: current status and potential solutions. *Microbiome* 2022;10:130. 10.1186/s40168-022-01320-0 35986393 PMC9392415

[74] Paver SF, Kent AD. Direct and context-dependent effects of light, temperature, and phytoplankton shape bacterial community composition. *Ecosphere* 2017;8:e01948. 10.1002/ecs2.1948

[75] Piccardi P, Vessman B, Mitri S. Toxicity drives facilitation between 4 bacterial species. *Proc Natl Acad Sci* 2019;116:15979–84. 10.1073/pnas.1906172116 31270235 PMC6690002

[76] Sundarraman D, Hay EA, Martins DM., et al. Higher-order interactions dampen pairwise competition in the zebrafish gut microbiome. *MBio* 2020;11:10.1128/mbio.01667-20. 10.1128/mbio.01667-20 PMC7554667 33051365

[77] Martiny JBH, Jones SE, Lennon JT., et al. Microbiomes in light of traits: a phylogenetic perspective. *Science (New York, NY)* 2015;350:aac9323. 10.1126/science.aac9323 26542581

[78] Morris JJ, Lenski RE, Zinser ER. The black queen hypothesis: evolution of dependencies through adaptive gene loss. *MBio* 2012;3:e00036–12. 10.1128/mBio.00036-12 22448042 PMC3315703

