# Supplementary material for: Environmental complexity shapes maintenance of bacterial diversity through context-dependent interactions among niche axes
Source: ISME J. 2026 Jun 11;20(1):wrag144. doi: 10.1093/ismejo/wrag144 (PMC13412393; doi:10.1093/ismejo/wrag144)
Supplement: MXC_Supplemental_Tables_and_Figures_v3_wrag144 [file mxc_supplemental_tables_and_figures_v3_wrag144.pdf]

Supplemental Tables and Figures

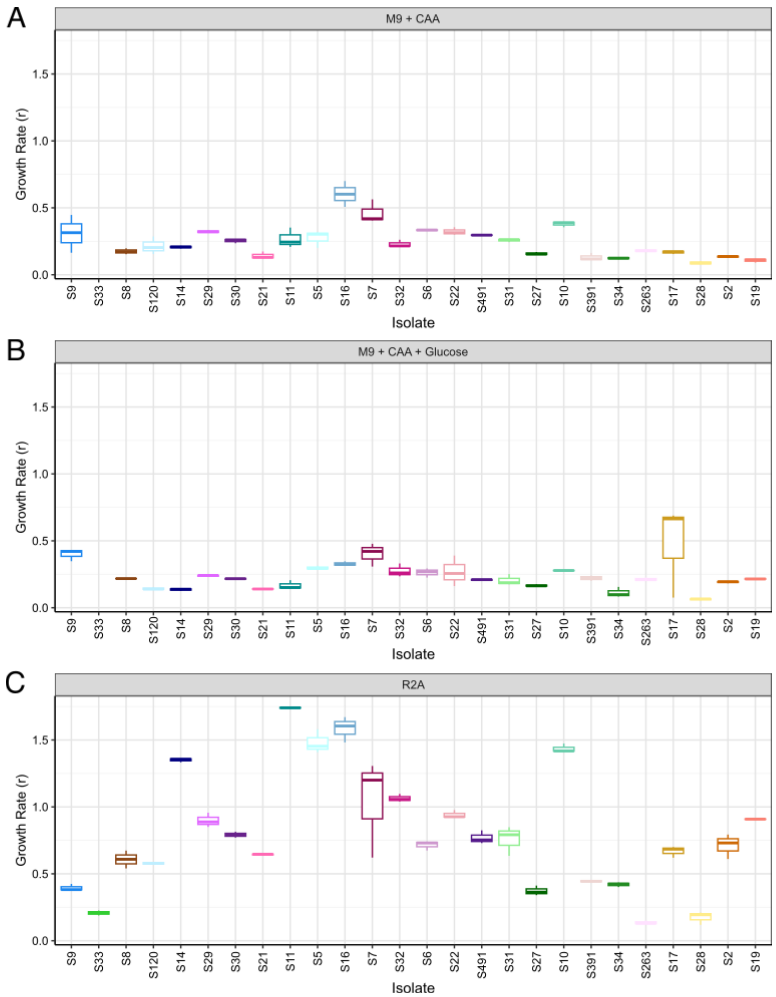

Supplemental Figure 1: Average growth rate for 3 replicates of each of the 26 bacterial isolates grown in (A) M9 + casamino acids (CAA) media, (B) M9 + casamino acids + glucose media or (C) R2A media. Growth curves were obtained by measuring OD_{600} every 5 min for 48 hours (R2A) or 96 hours (M9) on a SynergyMX plate reader and growth rates were calculated in R using the Growthcurver v0.3.1 package. Isolates are arranged according to their phylogenetic relationships as shown in Figure 5. Note that S33 did not grow in the M9 media.

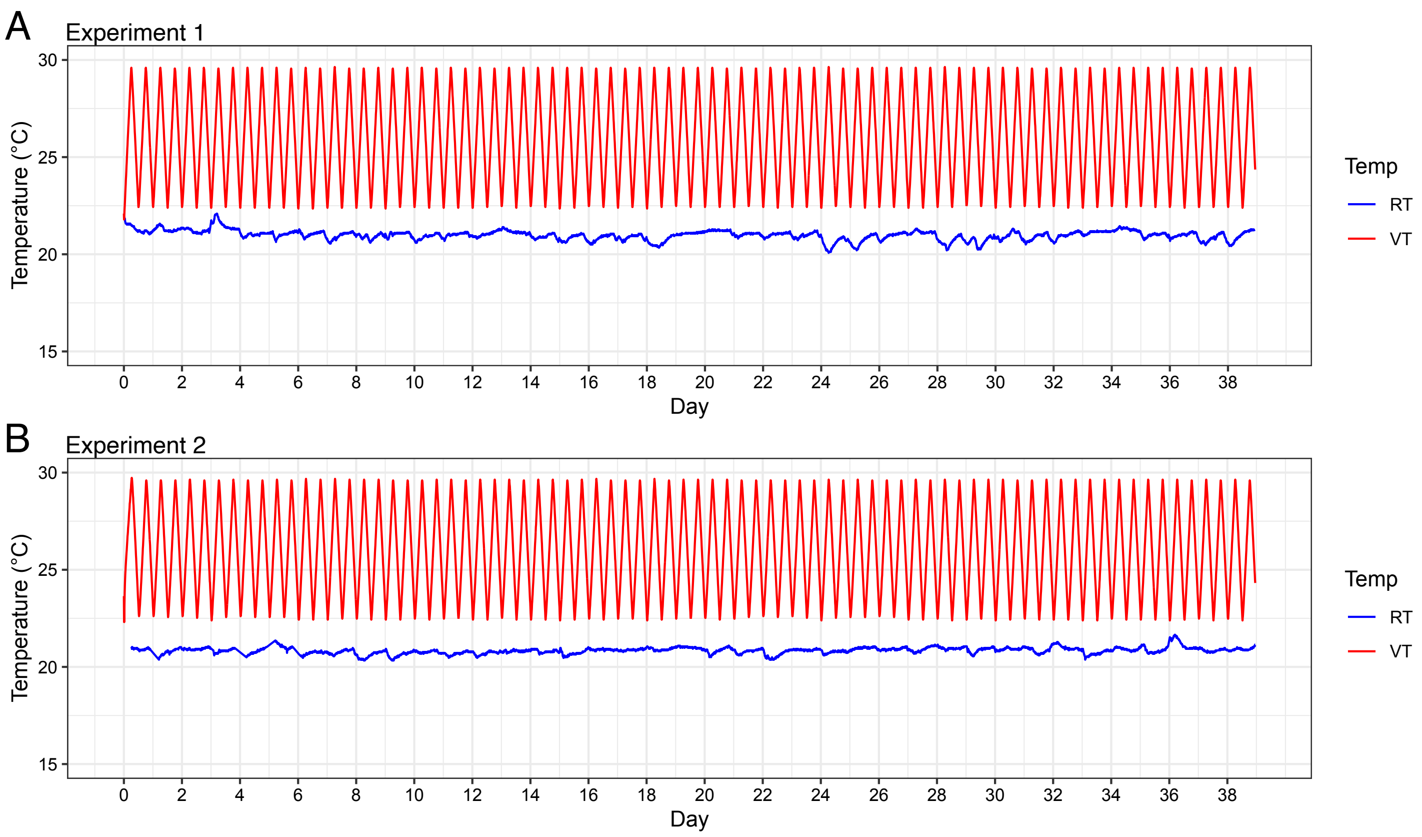

Supplemental Figure 2: Air temperature profiles for the two experiments: (A) Experiment 1 and (B) Experiment 2. Temperatures were measured every 10 minutes with a HOBO logger for the duration of each experiment. Constant room temperature conditions (RT) shown in blue and fluctuating variable temperature conditions (VT) shown in red.

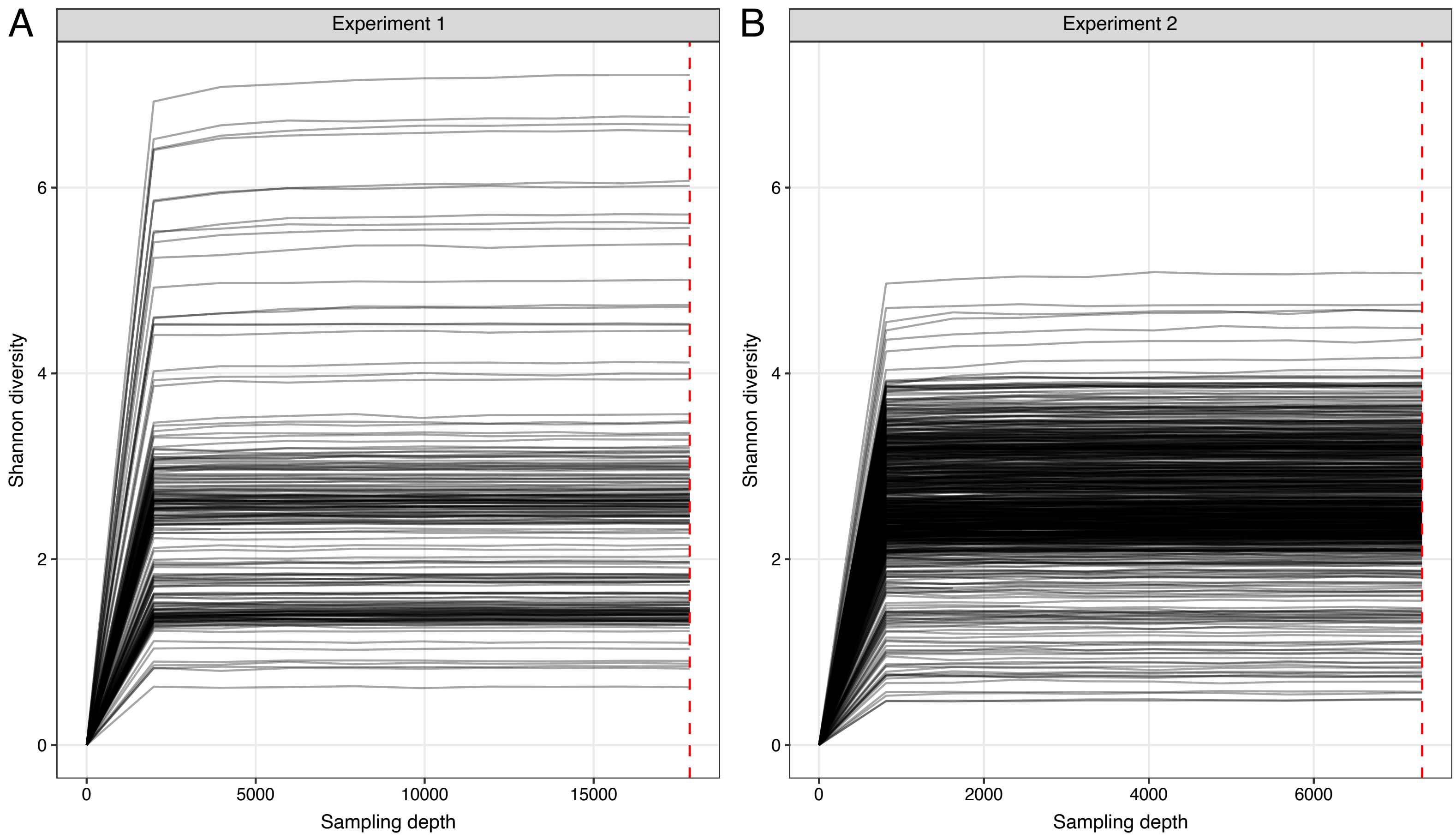

Supplemental Figure 3: Rarefaction curves for alpha diversity (Shannon index) across sequencing depths for (A) Experiment 1 and (B) Experiment 2. Rarefaction curves were generated from the unrarefied feature tables by subsampling reads across a range of sequencing depths. Each line represents an individual sample, with Shannon diversity plotted as a function of sequencing depth. Vertical dashed lines indicate the selected rarefaction depths for each experiment (17,841 reads per sample for Experiment 1 and 7,313 reads per sample for Experiment 2). Curves for both experiments approach a plateau at or below these thresholds, indicating that sequencing depth was sufficient to capture the majority of taxonomic diversity and supporting the use of these rarefaction depths.

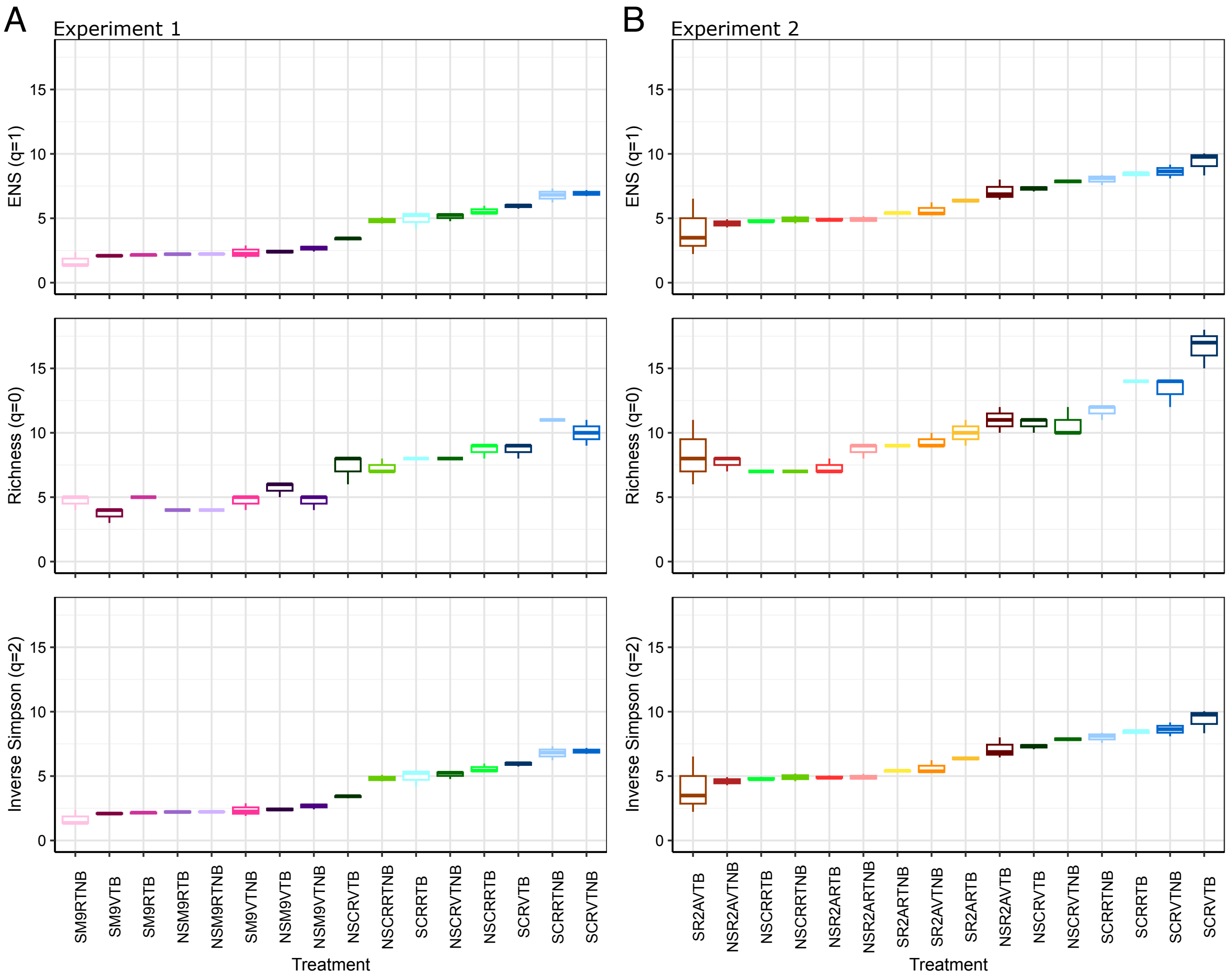

Supplemental Figure 4: Boxplots of Hill Numbers displayed as effective number of species (ENS), species richness, and inverse Simpson for (A) Experiment 1 and (B) Experiment 2 at the final experimental timepoint (day 39) for each treatment. Treatments are ordered by ENS value.

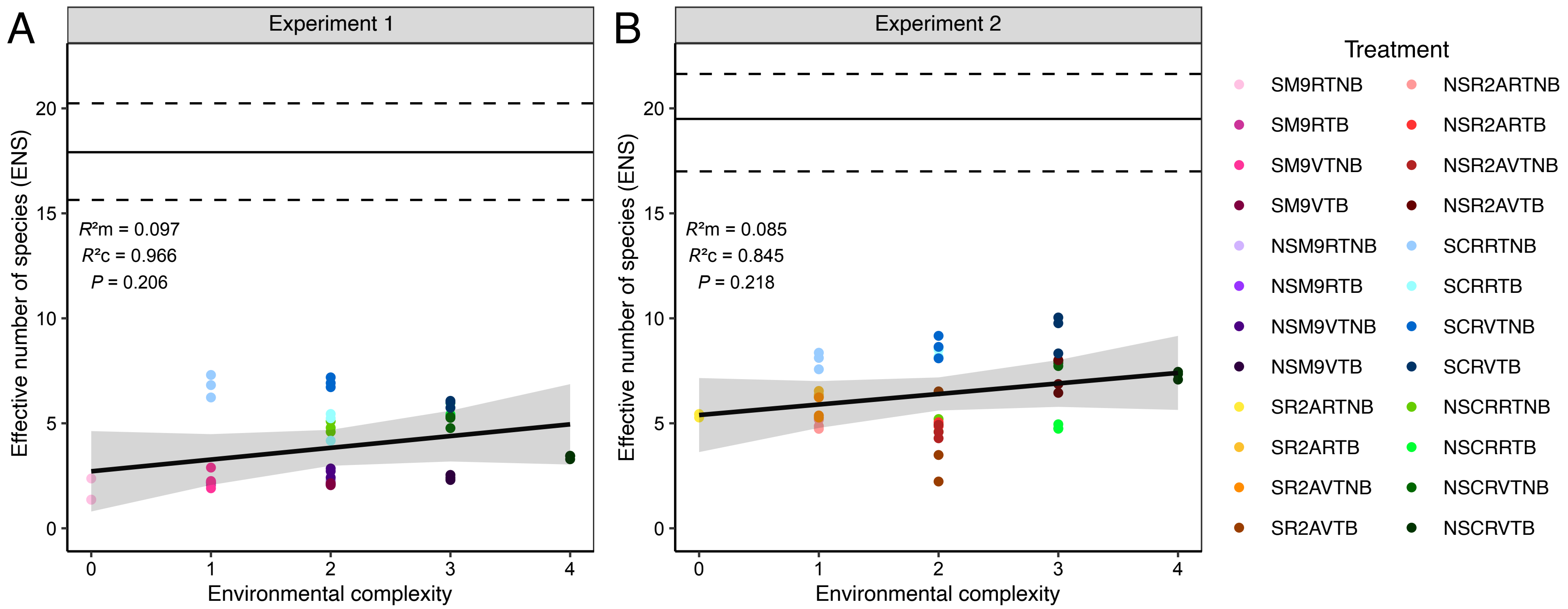

Supplemental Figure 5: Environmental complexity–diversity relationships relative to sampling-based null expectations. Points show the effective number of species (ENS; Hill number, q = 1) for individual communities across levels of environmental complexity in (A) Experiment 1 and (B) Experiment 2. Colors denote treatment identity. Solid black lines indicate fitted relationships from linear mixed-effects models, with shaded ribbons representing 95% confidence intervals of model predictions. Horizontal solid lines show the mean ENS expected under a null model based on random sampling from the initial species pool, and dashed lines indicate the corresponding 95% confidence intervals derived from 1,000 simulations. Marginal (R^2m) and conditional (R^2c) R^2 values are shown, along with the P-value associated with the fixed effect of environmental complexity in each mixed-effects model. R^2m represents variance explained by environmental complexity alone, whereas R^2c reflects variance explained by the full model including treatment-level variation.

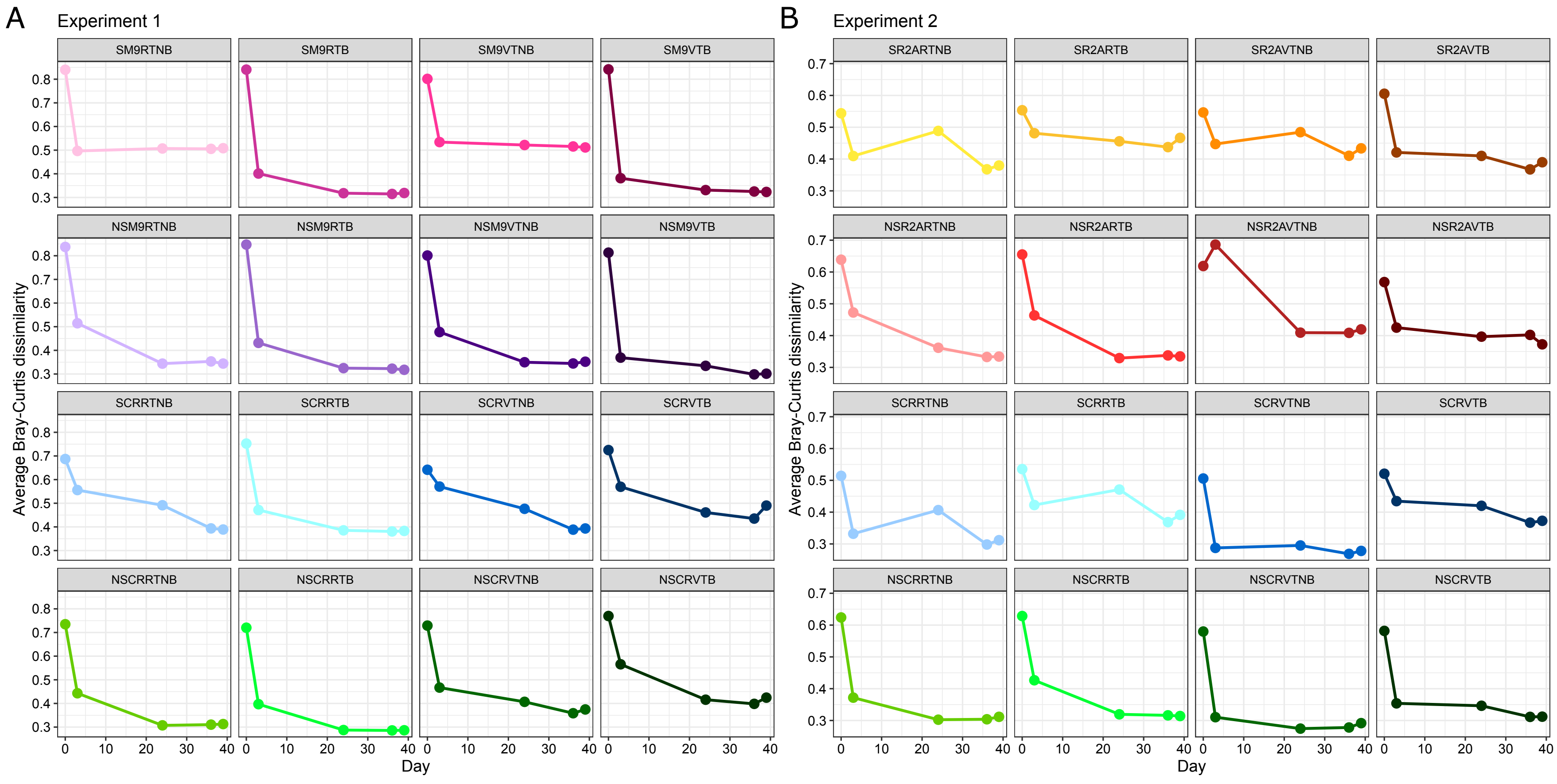

Supplemental Figure 6: Average Bray-Curtis dissimilarity values measured between samples from day 0, 3, 24, 36, 39 in (A) Experiment 1 and (B) Experiment 2 faceted by treatment.

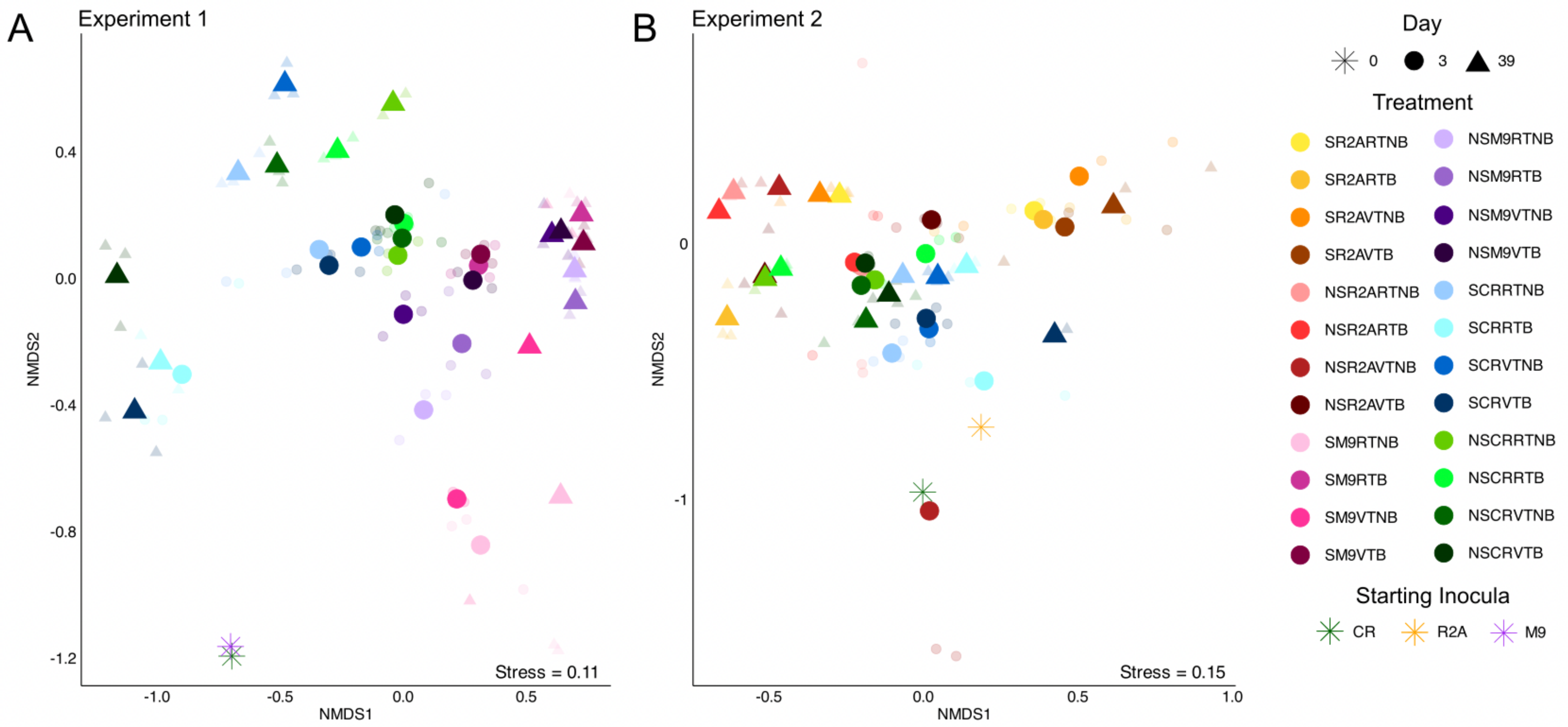

Supplemental Figure 7: Community composition across time and treatments. (A) and (B): Non-metric multidimensional scaling (NMDS) plots based on Bray-Curtis dissimilarity for (A) experiment 1 and (B) experiment 2 on day 0 (original community labeled by media used, shown as asterisks), 3 (3 days after initial incubation, circles), and 39 (final timepoint, triangles). Data centroids are shown in darker colors while individual sample data is displayed in lighter colors. The starting inocula used to create our experimental samples are shown as asterisks colored by their respective media type.

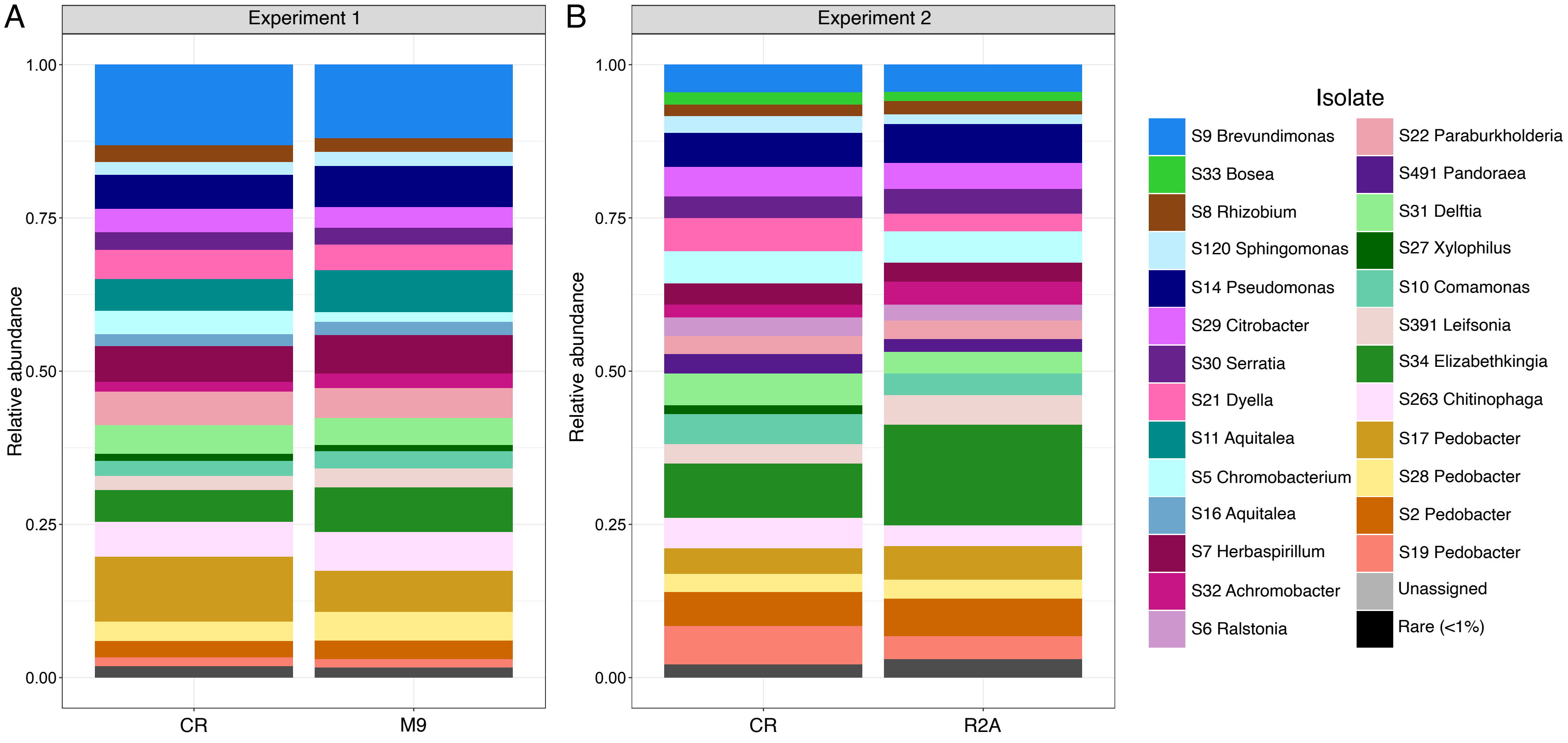

Supplemental Figure 8: Barplots from 16S rRNA gene amplicon sequencing data showing taxonomic community composition for the starting inoculum used in (A) Experiment 1 and (B) Experiment 2. The y-axis shows the relative abundance of each isolate in their respective media type (x-axis). CR = cricket-based media; M9 = M9 with casamino acids and glucose; R2A = Reasoner's 2A.

Supplemental Table 1: Taxonomy and genome information for each of the 26 strains utilized in experimental synthetic community. Taxonomic assignments were based on isolate 16S rRNA gene sequences obtained through Sanger sequencing and identified using BLAST. Taxonomic names were standardized according to the List of Prokaryotic Names with Standing in Nomenclature (LPSN), and the currently accepted names are reported here.

Strain Number	Strain ID	Sanger 16S rRNA Accession Number	Genome Accession Number	Phylum	Class	Order	Family	Genus	Top BLAST Match Sanger 16S rRNA	BLAST Query Cover	BLAST % Identity	Genome Completeness (%)	Genome Contamination (%)	Genome Assembled Coverage
S10	M05D5C33	PX401294	SAMN52183456	Proteobacteria	Gammaproteobacteria	Burkholderiales	Comamonadaceae	Comamonas	Comamonas testosteroni	98	97.19	99.85	0.31	101x
S11	M05D5GC01	PX401291	SAMN52183457	Proteobacteria	Gammaproteobacteria	Burkholderiales	Chromobacteriaceae	Aquitalea	Aquitalea pelogenes	96	97.06	99.15	0.85	10x
S120	M07D6C12	PX401308	SAMN52183458	Pseudomonadota	Alphaproteobacteria	Sphingomonadales	Sphingomonadaceae	Sphingomonas	Sphingomonas jeddahensis	90	95.98	99.64	1.81	85x
S14	M07D5C33	PX401315	SAMN52183459	Proteobacteria	Gammaproteobacteria	Pseudomonadales	Pseudomonadaceae	Pseudomonas	Pseudomonas protegens	97	96.88	99.38	1.43	102x
S16	M07D5GC13	PX401295	SAMN52183460	Proteobacteria	Gammaproteobacteria	Burkholderiales	Chromobacteriaceae	Aquitalea	Aquitalea aquatilis	96	94.78	99.04	0.43	26x
S17	M07D5GC17	PX401311	SAMN52183461	Bacteroidota	Bacteroidia	Sphingobacteriales	Sphingobacteriaceae	Pedobacter	Pedobacter chitinilyticus	86	96.6	97.45	0.48	60x
S19	M07D6GC08	PX401306	SAMN52183462	Bacteroidota	Bacteroidia	Sphingobacteriales	Sphingobacteriaceae	Pedobacter	Pedobacter nutrimenti	92	98.01	99.84	1.94	13x
S2	M03D5C18	PX401298	SAMN52183463	Bacteroidota	Bacteroidia	Sphingobacteriales	Sphingobacteriaceae	Pedobacter	Pedobacter nototheniae	93	99.01	97.61	0	102x
S21	M09D5C28	PX401293	SAMN52183464	Proteobacteria	Gammaproteobacteria	Xanthomonadales	Rhodanobacteraceae	Dyella	Frateuria defendens	88	98.06	100	0.85	30x
S22	M09D5GC07	PX401314	SAMN52183465	Proteobacteria	Gammaproteobacteria	Burkholderiales	Burkholderiaceae	Paraburkholderia	Paraburkholderia bannensis NBRC 103871	92	96.96	99.6	1.73	63x
S263	M03D5GC05	PX401300	SAMN52183466	Bacteriodota	Chitinophagia	Chitinophagales	Chitinophagaceae	Chitinophaga	Chitinophaga dinghuensis	95	97.43	100	0	58x
S27	M10D5C16	PX401299	SAMN52183467	Proteobacteria	Gammaproteobacteria	Burkholderiales	Comamonadaceae	Xylophilus	Xylophilus ampelinus	87	96.87	99.84	0	98x
S28	M10D5C22	PX401304	SAMN52183468	Bacteroidota	Bacteroidia	Sphingobacteriales	Sphingobacteriaceae	Pedobacter	Pedobacter xixiisoli	95	96.92	97.45	0	101x
S29	M10D5GC03	PX401297	SAMN52183469	Proteobacteria	Gammaproteobacteria	Enterobacterales	Enterobacteriaceae	Citrobacter	Citrobacter pasteurii	88	96.92	99.85	0.31	104x
S30	M10D5GC09	PX401305	SAMN52183470	Proteobacteria	Gammaproteobacteria	Enterobacterales	Yersiniaceae	Serratia	Serratia fonticola	98	97.27	99.94	0	104x
S31	M10D5GC11	PX401307	SAMN52183471	Proteobacteria	Gammaproteobacteria	Burkholderiales	Comamonadaceae	Delftia	Delftia acidovorans	92	97.45	99.85	0.23	101x
S32	M10D5GC19	PX401296	SAMN52183472	Proteobacteria	Gammaproteobacteria	Burkholderiales	Alcaligenaceae	Achromobacter	Achromobacter deleyi	91	98.05	99.53	0	85x
S33	M10D6C16	PX401312	SAMN52183473	Proteobacteria	Alphaproteobacteria	Rhizobiales	Alloboseaceae	Bosea	Bosea robiniae	78	97.3	99.58	1.1	96x
S34	M10D6GC05	PX401302	SAMN52183474	Bacteroidota	Bacteroidia	Flavobacteriales	Weeksellaceae	Elizabethkingia	Elizabethkingia bruuniana	95	97.26	100	2.15	105x

S391	M07D5C27	PX401310	SAMN52183475	Actinomycetota	Actinomycetes	Micrococcales	Microbacteriaceae	Leifsonia	Leifsonia naganoensis	97	96.75	99.49	0.76	54x
S491	M09D6C03	PX401301	SAMN52183476	Pseudomonadota	Betaproteobacteria	Burkholderiales	Burkholderiaceae	Pandorea	Pandoraea terrigena	84	97.95	99.5	0.69	101x
S5	M03D5GC02	PX401290	SAMN52183477	Proteobacteria	Gammaproteobacteria	Burkholderiales	Chromobacteriaceae	Chromobacterium	Chromobacterium phragmitis	98	93.94	98.61	1.31	100x
S6	M03D5GC15	PX401303	SAMN52183478	Proteobacteria	Gammaproteobacteria	Burkholderiales	Burkholderiaceae	Ralstonia	Ralstonia insidiosa	97	97.18	99.94	0.53	95x
S7	M03D5GC21	PX401313	SAMN52183479	Proteobacteria	Gammaproteobacteria	Burkholderiales	Oxalobacteriaceae	Herbaspirillum	Herbaspirillum frisingense	94	98.06	99.87	0.8	93x
S8	M03D6GC30	PX401292	SAMN52183480	Proteobacteria	Alphaproteobacteria	Rhizobiales	Rhizobiaceae	Rhizobium	Rhizobium lusitanum	95	97.48	99.44	2.91	48x
S9	M05D5C13	PX401309	SAMN52183481	Proteobacteria	Alphaproteobacteria	Caulobacterales	Caulobacteraceae	Brevundimonas	Brevundimonas vesicularis	90	98.47	99.68	0.65	27x
